# Positive and Negative Family Relationships Correlate With Mental Health Conditions -a Systematic Review and Meta-Analysis

**DOI:** 10.3389/phrs.2025.1607381

**Published:** 2025-07-21

**Authors:** Jutta Lindert, Sarah Arndt, Natalie Cook, Paul A. Bain, Ichiro Kawachi

**Affiliations:** ^1^ Department of Social Work and Health, University of Applied Sciences Emden/Leer, Emden, Germany; ^2^ Leeds and York Partnership NHS Foundation Trust, Leeds, United Kingdom; ^3^ Countway Library of Medicine, Harvard Medical School, Boston, MA, United States; ^4^ Department of Social and Behavioral Sciences, T.H. Chan School of Public Health, Harvard University, Boston, MA, United States

**Keywords:** family relationships, depression, anxiety, substance use, systematic review

## Abstract

**Objectives:**

We aim to investigate the association between family relationships and mental health conditions in adults aged 18+.

**Methods:**

We conducted a systematic review on associations of family relationships and mental health conditions by searching in databases MEDLINE, Embase, Web of Science Core Collection, PsycINFO, Sociological Abstracts (ProQuest), and PTSDPubs. We calculated the Pearson correlation coefficients and I2 statistics using a random-effects model. Additionally, we investigated publication bias using funnel plots.

**Results:**

Of the 3,707 records screened, 40 with n = 35,634 participants met the inclusion criteria (38.5% male, 59.5% female, mean age 39.57), were conducted mostly in North America (n = 27). Positive family relationships were investigated in 33 studies, negative relationships in 12 studies. Positive family relationships were not statistically significantly associated with depression [r = −0.071 (−0.256, 0.119), p = 0.463, anxiety r = 0.026 (−0.032, 0.084), p = 0.375] or alcohol abuse [r = 0.035 (−0.103, 0.0034), p=0.326]. Positive family relationships were statistically significantly associated with illicit drug use [r = 0.061 (0.025, 0.096), p = 0.001]. Negative family relationships were statistically significantly associated with anxiety [r = 0.075 (0.019, 0.130), p = 0.009], and with depression [r = 0.111 (0.033, 0.188), p = 0.005].

**Conclusion:**

Interventions reducing negative family relationships can potentially strengthen positive mental health.

## Introduction

Mental health conditions, including anxiety disorders and depression, are a public health challenge worldwide. Anxiety disorders affect approximately 3.6% and depression 4.4% of the global population [1]. In 2019, depressive conditions and disorders were the seventh leading cause of disability-adjusted life years (DALYs) [2]. These disorders affect individuals, their families [3], and societies [4]. However, the factors contributing to these conditions have not been fully understood. However, social relationships, including family relationships, play a crucial role in etiology and possible interventions for mental health conditions.

Family relationships are an essential component of human relationships [5]. Family relationships refer to the quality of family member relationships in cultural environments. Family relationships, however, are related to the context in which families live. An example of this culturally embedded family relationship is familismo, which consists of familial obligations and perceived support given and received by family members in Latino families [6]. Another example is the concept of filial piety, consisting of respect, love, and support by children in Asian families [7]. Family relationships, however, are associated with the health of family members [8–10].

It has been found that family relationships contribute to the incidence and prevalence of mental health conditions. Theories building on the empirical findings are the Family Systems Theory [11, 12], the Bio-Ecological Model [13], the Family Stress Model [14], the Developmental Systems Theory [15], the Attachment Theory [16, 17], the Circumplex Model [18], and the Bio-Behavioral Family Model (BBFM) [19]. The standard in these models is that positive family relationships are characterized by effective communication and adaptability, while conflicts and frequent arguments characterize negative family relationships. Furthermore, family relations relate to social capital, especially to bonding social capital. Family social capital has been associated with education outcomes [20] and children’s cognitive and social development [21, 22]. The social capital of families is measured mainly quantitatively without considering the nature and quality of the interaction [23, 24]. Yet studies investigating family social capital suggested that family relationships may contribute to depression, anxiety, and substance use in childhood and adolescence [25, 26].

Several mechanisms may link family relationships to mental health conditions. For example, conflicts can remove the feelings of social support. Despite evidence that family relationships are related to mental health conditions, there is limited understanding of their specific impact. To close this knowledge gap, we aim to 1) systematically synthesize studies on family relationships and mental health conditions in adults aged 18+ and 2) determine the specific impact of positive and negative family relationships.

## Methods

The review was registered with the International Prospective Register of Systematic Reviews, the Prospero Database (PROSPERO; identifier: CRD42019123240). We followed the Meta-analyses of Observational Studies in Epidemiology (MOOSE) checklist (Supplementary Table S1).

### Search Strategy

Cross-sectional or longitudinal studies examining the role of family relations (e.g., family cohesion, family conflict) in mental health outcomes were identified by searching the electronic databases MEDLINE (Ovid), Embase, Web of Science Core Collection (Clarivate), PsycINFO (EBSCO), Sociological Abstracts (ProQuest), and PTSDPubs (ProQuest) for studies that were published in peer-reviewed journals. The search strategy is presented in Supplementary Material S2. The citations and abstracts identified in the database search were saved and uploaded into COVIDENCE. The searches were developed by a subject expert (JL) in collaboration with an experienced medical librarian (PAB). Controlled vocabulary terms were included when available, and no date or language limits were applied. Search was last updated on 3rd November 2021 (Supplementary Material S3. Electronic Database Searches). Search terms for family relationships included social capital, social control, collective efficacy, and community participation, all within the context of the family (Supplementary Table S2). Additionally, we searched for the reference lists and citations of included articles.

### Eligibility Criteria

We used the following eligibility criteria: Studies were included if they were observational, the sample was at least 100+ adults aged 18+, and the measures provided opportunities for evaluating quantitative associations between family relationships and mental health conditions, alcohol abuse, or illicit drug use. Studies were excluded if they were not observational and if the participants were less than 18 years old.

### Family Models and Measurement Tools

The studies defined family relationships based on family models and concepts. We categorized negative family relationships with the following characteristics: lack of security in the family, expressed negativity towards a family member, relationship difficulties and positive family relationships, positive emotional bonds among family members, warmth, affective responsiveness, family`s adaptability, listening and speaking skills, reciprocity, respect, family’s ability to resolve problems and trust, support and confidence in family members (Table 1) (Supplementary Table S3)

**TABLE 1:** Family constructs and dimensions and effects of family constructs on mental health conditions (Worldwide 1998 - 2022).

First author	Construct and dimensions	Effects
Abe, 2004 [27]	Family harmony	Depression, anxiety in Japanese↓, no association in American students
Ai et al., 2014 [28]	Feeling of closeness to the family, social support: emotional support; negative family interactions	Anxiety↓; negative family interactions: Anxiety↑, Depression -
Ai et al., 2015 [29]	Feeling of closeness to the respondent’s family; b) Family conflict: Past conflict with the respondent’s families	Closeness: −; Negative family interactions: Depression↑
Bakhtiari et al., 2017 [30]	Emotional bonding between family members, family conflict: not meeting parental expectations	Depression-
Bert et al., 2022 [31]	Emotional bonds	Suicidal ideation↓
Bert et al., 2020 [32]	Emotional bonds	Depression↓
Caetano et al. 2017 [33], Caetano et al., 2018 [34]; Caetano et al., 2019 [35]	Sharing of shared values and beliefs, trust among family members, loyalty to and pride in the family, sharing time with family, closeness to family members	Low closeness: AUD, drug use↑; low/moderate FC: Depression↑; binge drinking -
Cano et al., 2018 [36]	The feeling of togetherness in the family	AUD among males↓
Carris et al., 1998 [37]	Family rigidity: Strict rules in response to situational and developmental stress	Family rigidity: Suicidal ideation↑
Darghouth et al., 2015 [38]	Family closeness and communication within the family, connections with and feelings of support by relatives, family conflicts	Psychological distress -; conflicts: Psychological distress↑
Diamond et al., 2008 [39]	Family bonds, family adaptability	Family bonds, adaptability: Alcohol/cannabis/drug use↓, frequency -
Dillon et al., 2012 [40]	Emotional bonds between family members, close relationships with family members throughout life, including loyalty, reciprocity, solidarity	Alcohol//drug use↓
Escobedo et al., 2018 [41]	Respect, fidelity, interdependence	Familism: Binge drinking; respect: binge drinking↓
Guassi Moreira and Telzer, 2015 [42]	Quality of relationship/communication, mutual trust	Relationship quality: Depression↓
Guo et al., 2015 [43]	Respect, shared values and beliefs, trust, and confidence in each other; feeling loyal to and pride of family; express feelings within the family, spending free time together, family closeness; family support	Respect, shared values: depression↓, family support: anxiety, mood disorders -
Guo et al., 2018 [44]	Spouse/family support, filial piety (respect, care, checking in and on members, pleasure, obedience, and financial support they receive)	Family support -, Filial piety: depression/anxiety↓
Gyasi et al., 2019 [45]	Frequency of family contacts	Frequency of family contacts: Distress -
Joel Wong et al., 2012 [46]	Belonging: loyalty to one’s family, filial piety, fulfilling familial obligations, maintaining harmonious family relationships	Belonging: Suicidal ideation↓
Kwon, 2020 [47]	Closeness (spending free time together, closeness and togetherness), family conflict (feeling too close to family, family interfering with own goals), spouse/partner support (favorable interactions), - strain (criticism by partner)	Family conflict; spouse/partner strain: Psychological distress ↑, spousal/partner support: distress -
Leong et al., 2013 [48]	Closeness, respect, sharing values, spending time together, working well together, trust, loyalty; family conflict: family interfering with own goals, loneliness because of lack of family unity	Latinos: Closeness: depression↓, low family conflict: anxiety, depression/SUD↓, Asians: FC: anxiety↑, high conflict: anxiety, depression↑, low conflict: anxiety, depression↓
Levesque and Quesnel-Vallée, 2019 [49]	Family social capital: Family ties (strength of the relationship to family members)	Fair/poor self-rated mental health↓, binge drinking -
Litwin and Shiovitz-Ezra, 2011 [50]	Family structure (higher number of children, higher number of close relatives)	Anxiety↓
Luna et al., 2020 [51]		
Markwick et al., 2015 [27]	Inability to get help from family	Psychological distress↑
Morimoto and Sharma, 2004 [52]	Positive emotional relationships, time spent together, shared interests and activities, parental verbal aggression	Depression↓, parental verbal aggression: Depression↑
Nam et al., 2016 [53]	The ability of the family to maintain strong emotional bonds between family members, the degree to which the family can cope with changes, how flexible the family system is in facing changes and situational stress	Depression↓
Park et al., 2014 [54]	Family emotional bonds, spending time together, the importance of closeness and togetherness in the family, and family conflict	Depression↓, family conflict: Depression↑
Park, 2017 [55]	Family size	More family members: Depression↓
Priest and Denton, 2012 [56]	Family unity, family conflicts: discord, prioritizing familial obligations, honoring the family, and using the family as referents for the definition of self	Unity: GAD↓, PTSD - Discord: GAD, anxiety, PTSD ↑
Rivera et al., 2008 [57]	Emotional closeness bonds that family members have toward one another	Psychological distress↓
Savage and Mezuk, 2014 [58]	Emotional closeness/bonds, family conflict: argument with the family	AUD/SUD: -, family conflict: AUD/DUD: -
Wang et al., 2021 [59]	Negative family interactions (family members making demands, criticize, take advantage of you)	Depression in whites↑
Westrick et al., 2021 [60]	Doing things together, help and support each other	Alcohol use severity↓
Xie et al., 2021 [61]	Positive family relationships (cohesion, conflicts)	Anxiety, depression↓; family conflict: anxiety, depression↑
Yang and Mills, 2008 [62]	Emotional attachment among family members, feelings of intimacy toward one’s family, adaptability	Depression -

### Study Selection

We selected the studies based on eligibility criteria (Supplementary Figure S1). After removing duplicates, we screened the titles and abstracts of the remaining articles for eligibility. At least two authors of the authors (MN, SA, or NC) independently reviewed each full-text manuscript. Like in the abstract processing, we resolved disagreements by discussing the study selection criteria with the lead author (JL). Based on the study eligibility criteria, we included 40 articles in this systematic review.

### Data Extraction

First, we developed a standardized data extraction template with the basic study information (year of publication, sample size and characteristics, study design), the theoretical framework for family relationships (if provided), methodology (e.g., assessment of family relationships), the outcomes (e.g., depression, anxiety, substance use, distress; Supplementary Table S2), and adjusted measures of associations between family relationships and mental health conditions, alcohol abuse and illicit drug use. Using the standardized template, two authors independently extracted data (SA, NC). We synthesized and described this information in Supplementary Table S3.

### Risk of Bias

We used the Critical Appraisal Skills Programme (CASP) (Supplementary Table S3) to evaluate the risk of bias. The overall appraisal domains were a) sample, b) control/comparison, c) exposure assessment, d) outcome measures, and e) potential confounders. Each domain is broken down into sub-criteria, such as representativeness of the population, clearly stated sampling method, and validity and reliability of measurements. Two researchers (SA and NC) independently conducted the risk of bias assessment. The two researchers resolved disagreements in the assessment by double-checking the items in question. In case of doubt, a third researcher (JL) provided help.

### Data Analysis

Metanalyses were conducted using Comprehensive Meta-Analysis version 3.3070 software (CMA). We calculated the Pearson Correlation Coefficient r. In case studies reported other effect size measures, such as odds ratios (ORs), the effects were converted to rs [63]. Only one effect size or study was used to ensure independence among effect sizes. The correlation coefficients from each study were converted to the Fisher’s Z scale to obtain a normal sampling distribution [64, 65]. These transformed Fisher’s Z values were subsequently re-converted to rs [63]. Study effect sizes were weighted by the inverse of their variance [65] before combining [66]. Data on the association between exposures and mental distress is analyzed but not shown.

Subsequently, random effects models were used to combine the effect sizes across studies, generating a weighted mean effect size and 95% confidence intervals for the overall association between positive and negative family relationships and mental health conditions. Weighted mean effect sizes with confidence intervals that did not include zero were considered to be statistically significant (i.e., Z-test with p values <0.05), with Cohen’s d [67]. Guidelines were used to interpret the magnitude of the mean correlation coefficient for significant associations (r ≈ 0.10 as small, r ≈ 0.30 as medium, and >0.50 as substantial).

Heterogeneity tests were conducted using the *I* ^2^ and the Q statistics, determining how much variation exists between studies because of study differences and not because of chance. The Q statistic assessed whether the pooled effect sizes had a homogeneous distribution across studies; p values of <0.05 indicated significant heterogeneity of study effect sizes due to sources other than random sampling error [68, 69]. Furthermore, we performed sensitivity analyses to assess whether the meta-analysis results were influenced by the studies that evaluated “partner support” (the process of responding with helping behavioral as well as psychological acts to a difficulty or a problem of one’s partner in a couple of relationships) in addition to “family support” (the process of responding with helping behavioral as well as psychological acts to a difficulty or a problem of in a family).

Additionally, we investigated publication bias using both the rank correlation test, Kendall’s tau (τ) [70], and Egger’s linear regression intercept test [71]. We conducted all analyses with Comprehensive Meta-Analysis Version 4.

### Risk of Bias Evaluation

We identified thirty studies with a low risk of bias, seven with a medium, and three with a high risk of bias (Supplementary Table S1). A potential source of bias was the small sample sizes. Most of the studies did not report power calculations. Further risk of bias was associated with the study design. Most studies (37 out of 40) used a cross-sectional design, which cannot tease out temporality, i.e., risk of reverse causation.

## Results

**FIGURE 1:**
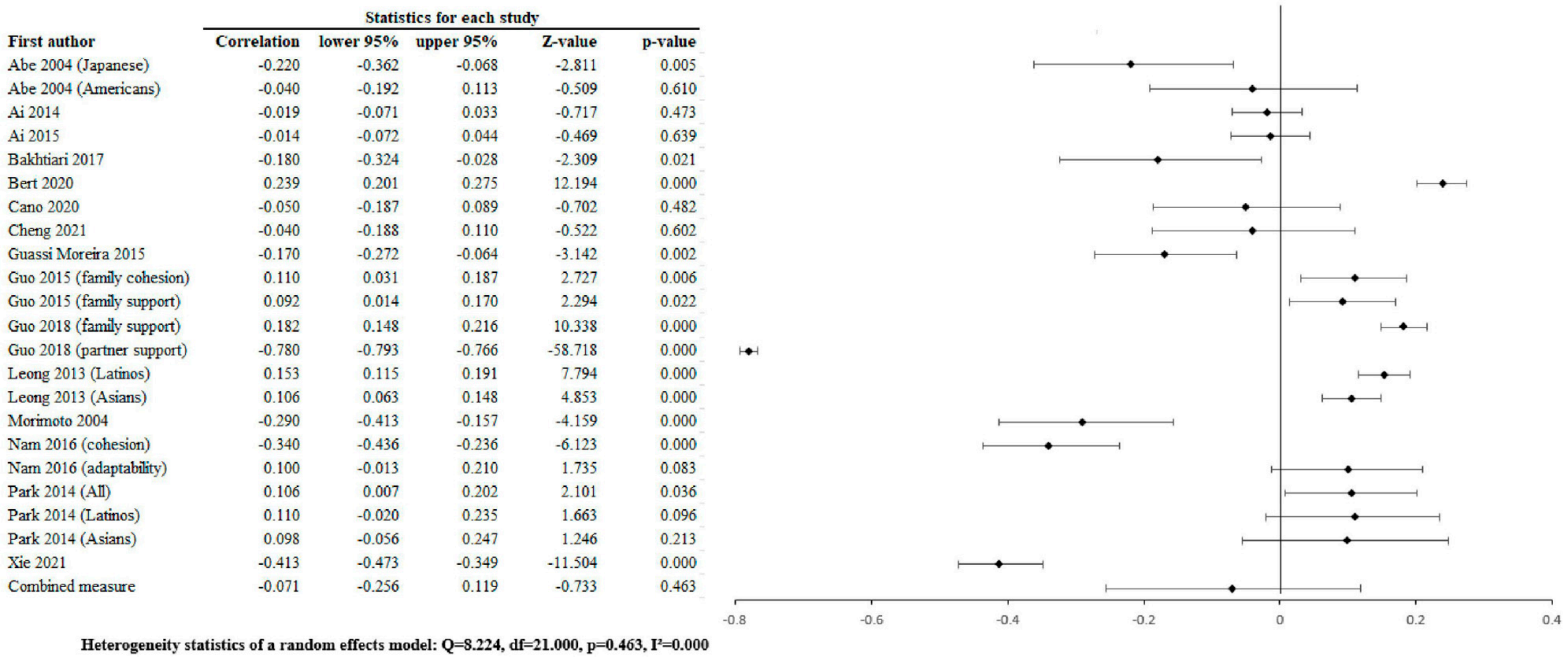
Positive family relatiions and depression (Worldwide 2004 - 2021).

Electronic database searching produced 3,707 unique records, of which 362 were selected for full-text review. Forty studies were included in our analysis (Figure 1). These studies reported measures of association between positive or negative family relationships with symptoms of depression, anxiety, or alcohol or illicit substance abuse (Supplementary Table S4).

### Description of Studies

Most studies were cross-sectional (n = 37) or cohort (n = 3) studies [42, 55, 72] (Supplementary Figure S1). Sample sizes varied from n = 161 [51] to n = 9,890 participants [49]. In total, we provide results for n = 32,982 individuals (mean sample size: n = 1,320 (SD = 285.24), 59.5% female; 38.5% male. Most studies were conducted in the United States (USA) (n = 28, 75%) [27–48, 50, 52–54, 56–61, 72–75]; others in Australia [34], Canada [74], Ghana [62], Israel [76], Italy [77, 78], Mexico [33], South Korea [53], or Taiwan [62] (Supplementary Table S4).

Various tools related to the models were used to assess family relationships (Supplementary Table S5). The most used tool was the Family Adaptability and Cohesion Evaluation Scale (FACES) [18] (n = 15, 37.5%), followed by the Family Environment Scale (FES) (43) (n = 5, 12.5%) and the Family Functioning Scale (FFS) (n = 1) [79]. Additionally, measures developed for the studies were used to assess positive and negative family relationships. Key details of the studies are listed in Supplementary Table S4.

### Risk of Bias Analysis

We found that most studies were at moderate risk of bias. Participants were mainly purposively recruited. Most studies provided detailed inclusion and exclusion criteria. Sample sizes varied, and most studies did not report response rates (Supplementary Tables S4–S7).

### Study Synthesis

**FIGURE 2:**
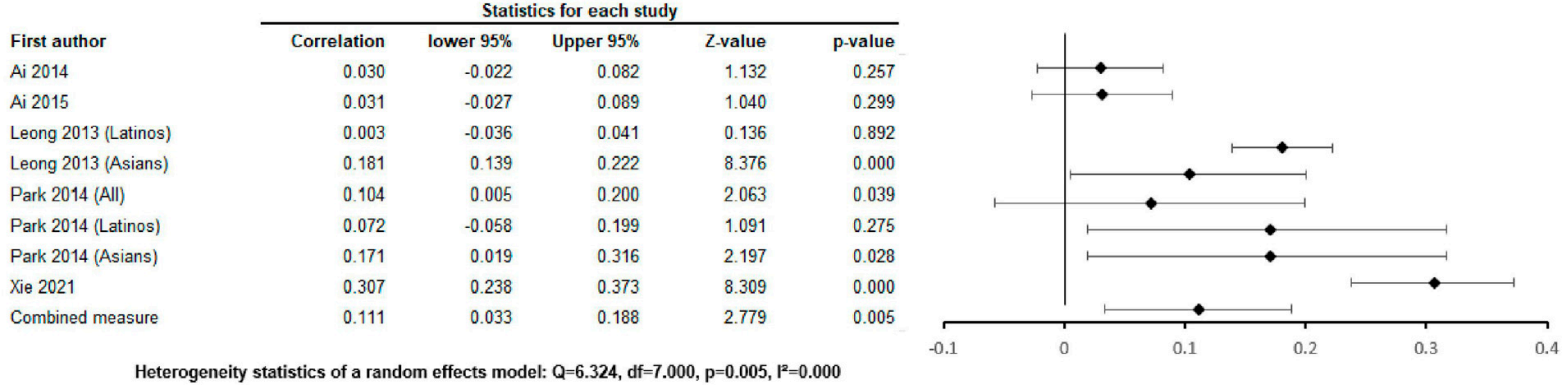
Negative family relations and depression (Worldwide 2013 - 2021).

**FIGURE 3:**
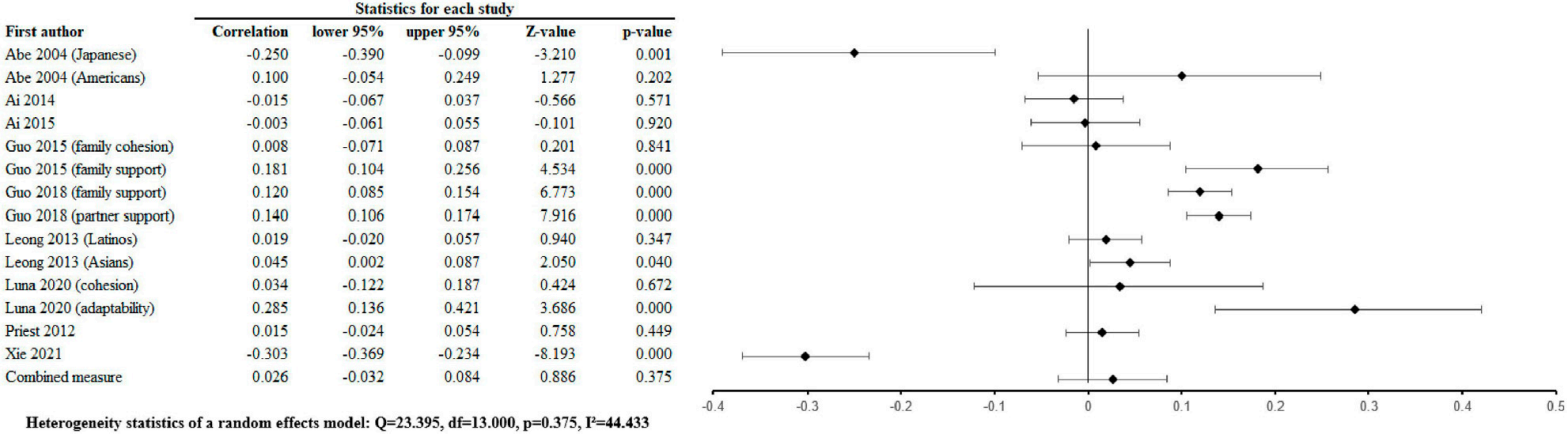
Positive family relations and anxiety (Worldwide 2004 - 2021).

**FIGURE 4:**
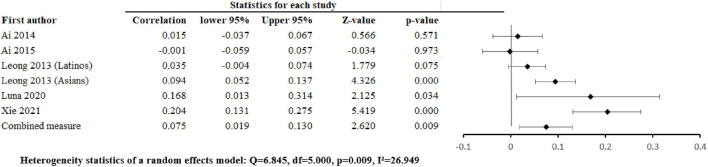
Negative family relations and anxiety (Worldwide 2014 - 2021).

The pooled associations of positive family relationships with depression were r = –0.071 [–0.256, 0.119] (Figure 2), with anxiety (Figure 3) were r =0.026 [-0.032, 0.084], p = 0.375, p = 0.463, with alcohol abuse r = 0.035 [–0.103, 0.0034], p = 0.326 (Supplementary Figure S5), and with illicit drug abuse r = 0.061 [0.025, 0.096], p = 0.001 (Supplementary Figure S7). Negative family relationships were statistically signicantly associated with depression (r =0.111 [0.033, 0.188], p = 0.005) (Figure 2) and anxiety (r = 0.075 [0.019, 0.130], p = 0.009) (Figure 4).

Analyzing the heterogeneity of studies, there was no evidence of heterogeneity in the studies related to positive family relationships and mental health conditions (Q = 6.324, p = 0.005, I2 = 0.0000) (Supplementary Figures S2, S3).

### Publication Bias

There was no evidence of publication bias by applying visual inspection of the funnel plots (Supplementary Figure S4) and analyzing Egger’s regression intercept (mental health conditions: t = 2, 188, p = 0.034).

### Sensitivity Analyses

To better understand whether partner relationships or gender moderate the effects of positive and negative family relationships on depression, anxiety, overall mental health conditions, and substance use, we investigated whether positive family relationships vary in case partner relationships are included or not in the analyses. However, these associations were similar in magnitude to the original overall correlation coefficients. The sensitivity analyses suggest that family support without partner support had no additional effect.

## Discussion

This is the first meta-analysis that provides comprehensively synthesized estimates of the relationship between positive and negative family relationships and adults' anxiety, depression, alcohol abuse, and illicit drug use. Integrating the results of these 40 studies shows that negative family relationships, including insecure attachment, parent-related family conflict, and negative communication styles, are each positively associated with anxiety and depression. While identification of this association is the first stage, only after understanding its origins can effective solutions be sought to help reduce depression and anxiety.

The findings align with family models Supplementary Table S3, especially the attachment theory, outcome-oriented, process-oriented models, and the Thriving Through Relationships model. The attachment model, which suggests that a lack of secure belonging is the center of mental health conditions, could be helpful in this context; precisely, negative attachments convey a person’s belief that others are not interested in relationships. Similarly, continuous and frequent family conflicts convey to individuals that others are primarily a source of unpleasant feelings and insecurity. Therefore, people receiving these messages might develop mental health conditions. However, positive family relationships were not related to mental health conditions in our review. In the outcome-oriented model [18, 80], families with conflicts tend to be rigid or chaotic and have difficulties adapting to crises. Conflicts lead to more stress for family members, which subsequently may affect mental health conditions. The process-oriented theories of the family suggest that if families are not able to effectively deal with events, chronic dysfunction and various mental disorders (e.g., depression, anxiety) may emerge. The Thriving Through Relationships Model suggests that to have a positive influence, family relationships must provide responsive support. Responsive support means that the support provided is responsive to the needs of the family members receiving the support. Conversely, unresponsive family relationships may cause stress. The Social Capital of Families theory has focused so far on children’s outcomes. We add knowledge on adults' outcomes. Our findings align with empirical studies suggest that not all types of social relationships positively influence mental health [76, 77]. While some family relationships promote mental health through strengthening safety and responding to the fundamental need to belong, family relationships with conflicts may contribute to feelings of not belonging.

We found heterogeneity in the associations between family relationships and mental health conditions. Our analysis suggests that positive family relationships are not significantly related to depression, anxiety, or alcohol abuse. We found, however, that positive family relations were positively associated with reduced illicit drug use. This finding supports recent studies suggesting that family cohesion during adolescence is associated with various self-regulatory outcomes. Lack of self-regulation may be associated with using illicit drugs.

The findings suggest that conflict relationships are associated with depression and anxiety. As we could not calculate the relationships between conflicts between alcohol abuse and illicit drug use, we can provide findings on this association. These findings are consistent with studies suggesting that the quality of relationships plays a significant role in mental health conditions in adult life. A reason for the heterogeneity in study findings might be that the questionnaires used to evaluate exposure and outcome were not validated for use in each country. Additionally, it might be that the questionnaires were not adequately translated.

The study has some strengths: the large sample size, the use of various databases, and sensitivity analyses. The five electronic databases in which the search was conducted were carefully chosen based on guidance from an experienced university librarian (PB). However, several limitations deserve to be mentioned. This review includes data from ten countries, particularly high-income countries. However, including studies using various assessment tools contributes to the observed heterogeneity. It might be that using assessment tools that have been validated and not thoroughly validated contributes to heterogeneity in study results. However, validating family cohesion and family support assessment tools will be difficult as no “gold standard” for these tools exists. A random-effects meta-analysis was adopted to account for the between-study heterogeneity; however, random effects meta-analysis gives a higher weight to smaller studies. We did not examine whether instruments to measure family relationships affected the relationship between positive and negative family relationships and mental health conditions because the variety of instruments used was too large.

Despite the limitations, our review is, to the best of our knowledge, this study is the first to conduct a meta-analysis of the association between negative and positive family relationships and mental health conditions, revealing that negative family relationships are relevant to the development or maintenance of mental health conditions. Our findings suggest that future research should focus on specific quality elements of family relationships. In clinical practice, given that negative family relationships are negatively related to mental health conditions in adults, it may be valuable to develop specific toolboxes to identify families at risk for damaging relationships.

Our review suggests that it is essential to recognize that conflicts in family relationships can negatively impact mental health. Based on the results of our review, we argue that interventions reducing the impact of conflicts in family relationships hold the potential to reduce mental health conditions. In this conclusion, we agree with other researchers who have similarly argued for the relevance of public mental health interventions developed to improve social and especially family relationships [81]. In particular, the results of our study cement the value of working with families to improve the mental health of family members. Further research is needed to confirm this pattern. This research might consider the quality of relationships in different cultures and different age groups. Our findings suggest that more research is needed on the impact of negative family relationships instead of positive ones.
