# Supplementary material for: Positive and Negative Family Relationships Correlate With Mental Health Conditions -a Systematic Review and Meta-Analysis
Source: Public Health Rev. 2025 Jul 21;46:1607381. doi: 10.3389/phrs.2025.1607381 (PMC12320053; doi:10.3389/phrs.2025.1607381)
Supplement: Supplementary file 1 [file DataSheet1.docx]

**Supplementary Online Material**

**Supplemental Material 1. Table 1: MOOSE Checklist for Meta-analyses of Observational Studies**

**Supplemental Material 2. Electronic Database Searches**

**Supplemental Material 3. Table 2: Search terms for family relationships**

**Supplemental Material 4. Figure 1: Flow chart**

**Supplemental Material 5. Table 3: Models, summary of model, measurement tools**

**Supplemental Material 6. Table 4. Study characteristics (authors, country, study design, sample, exposure, outcome, covariates, results)**

**Supplemental Material 7. Table 5: Mental health conditions, measures to assess mental health conditions and studies included in the systematic review**

**Supplemental Material 8. Table 6: Risk of Bias of cross-sectional studies in the systematic review on family relationships and mental health outcomes**

**Supplemental Material 9. Table 7: Risk of Bias of cohort studies in the systematic review on family relationships and mental health outcomes**

**Supplemental Material 10. Figure 2. Funnel plot for studies on positive family relationships and depression**

**Supplemental Material 11. Figure 3: Funnel plot for studies on negative family relationships and depression**

**Supplemental Material 12. Figure 4: Funnel plot for studies on positive family relationships and anxiety**

**Supplemental Material 13. Figure 5: Funnel plot for studies on negative family relationships and anxiety**

**Supplemental Material 14. Figure 6: Associations between positive family relationships and alcohol abuse**

**Supplemental Material 15. Figure 7: Funnel plot for studies on positive family**

**Supplemental Material 16. Figure 8: Associations between positive family relationships and use of illicit substances**

**Supplemental Material 17. Figure 9: Funnel plot for studies on positive family and illicit substances use**

**Supplemental Material 18. References**

**Supplementary Material**

**Supplementary material 1**

**Table 1. MOOSE Checklist for Meta-analyses of Observational Studies**

| **Item No** | **Recommendation** | **Reported in section** |
| --- | --- | --- |
| Reporting of background should include | | |
| 1 | Problem definition | Abstract, background |
| 2 | Hypothesis statement | - |
| 3 | Description of study outcome(s) | Abstract, methods |
| 4 | Type of exposure or intervention used | Abstract, methods |
| 5 | Type of study designs used | Abstract, methods |
| 6 | Study population | Abstract, methods |
| Reporting of search strategy should include | | |
| 7 | Qualifications of searchers (eg, librarians and investigators) | Title page |
| 8 | Search strategy, including time period included in the synthesis and key words | Supplementary material |
| 9 | Effort to include all available studies, including contact with authors | Methods |
| 10 | Databases and registries searched | Methods |
| 11 | Search software used, name and version, including special features used (eg, explosion) | Methods |
| 12 | Use of hand searching (eg, reference lists of obtained articles) | Methods |
| 13 | List of citations located and those excluded, including justification | Methods, Figure 1 Supplementary material |
| 14 | Method of addressing articles published in languages other than English | Methods |
| 15 | Method of handling abstracts and unpublished studies | Methods |
| 16 | Description of any contact with authors | Methods |
| 17 | Description of relevance or appropriateness of studies assembled for assessing the hypothesis to be tested | - |
| 18 | Rationale for the selection and coding of data (eg, sound clinical principles or convenience) | Methods |
| 19 | Documentation of how data were classified and coded (eg, multiple raters, blinding and interrater reliability) | Methods |
| 20 | Assessment of confounding (eg, comparability of cases and controls in studies where appropriate) | Methods |
| 21 | Assessment of study quality, including blinding of quality assessors, stratification or regression on possible predictors of study results | Methods, Supplementary material |
| 22 | Assessment of heterogeneity | Methods, Supplementary Material, Figures |
| 23 | Description of statistical methods (eg, complete description of fixed or random effects models, justification of whether the chosen models account for predictors of study results, dose-response models, or cumulative meta-analysis in sufficient detail to be replicated) | Methods |
| 24 | Provision of appropriate tables and graphics | Tables 2-7, Figures 2-7 |
| 25 | Graphic summarizing individual study estimates and overall estimate | Figures 6, 8 |
| 26 | Table giving descriptive information for each study included | Table 2 |
| 27 | Results of sensitivity testing (eg, subgroup analysis) | Supplementary material |
| 28 | Indication of statistical uncertainty of findings | Results, Supplementary material |
| **Item No** | **Recommendation** | **Reported on Page No** |
| 29 | Quantitative assessment of bias (eg, publication bias) | Results, Supplementary material |
| 30 | Justification for exclusion (eg, exclusion of non-English language citations) | Methods |
| 31 | Assessment of quality of included studies | Results, Supplementary material |
| 32 | Consideration of alternative explanations for observed results | Discussion |
| 33 | Generalization of the conclusions (ie, appropriate for the data presented and within the domain of the literature review) | Discussion |
| 34 | Guidelines for future research | - |
| 35 | Disclosure of funding source | 20 |

Stroup DF, Berlin JA, Morton SC, et al. Meta-analysis of Observational Studies in Epidemiology. A Proposal for Reporting. *JAMA*. 2000;283(15:2008- 2012).

**Supplementary Material 2**

**Electronic Database Searches**

MEDLINE (Ovid)

Ovid MEDLINE(R and Epub Ahead of Print, In-Process, In-Data-Review & Other Non-Indexed Citations, Daily and Versions(R 1946 to November 02, 2021

November 3, 2021

899 Records

1. (exp social capital/ OR (social adj (capital OR cohesion OR control OR organi?ation.ab,ti OR informal control.ab,ti OR collective efficacy.ab,ti) OR (family adj2 (cohesion OR organi#ation or disorganiation .ab,ti OR (community OR neighborhood adj2 (cohesion OR participation.ab,ti)

2. (exp family/ OR exp family relations/ OR family.ab,ti OR families.ab,ti

3. (exp depressive disorder/ OR exp depression/ OR exp anxiety disorders/ OR depression.ab,ti OR depressive.ab,ti OR anxiety.ab,ti OR panic.ab,ti OR obsessive.ab,ti OR ocd.ab,ti OR exp stress disorders, traumatic/ OR (stress adj (disorder* OR symptom* OR syndrome*.ab,ti OR ((traumatic OR posttraumatic adj (stress* OR psycho* OR neurosis.ab,ti OR ptsd.ab,ti OR exp anger/ OR exp hostility/ OR anger.ab,ti OR rage.ab,ti OR hostility.ab,ti OR exp substance-related disorders/ OR exp alcoholics/ OR exp drug users/ OR alcohol.ab,ti OR alcoholism*.ab,ti OR alcoholic*.ab,ti OR heroin.ab,ti OR opiate*.ab,ti OR opioid*.ab,ti OR opium.ab,ti OR narcotic*.ab,ti OR cannabis.ab,ti OR marijuana.ab,ti OR hashish.ab,ti OR ganja.ab,ti OR cocaine.ab,ti OR methaqualone.ab,ti OR ephedrine.ab,ti OR acetic anhydride.ab,ti OR amphetamine*.ab,ti OR inhalant*.ab,ti OR glue.ab,ti OR ((drug* OR substance* adj1 (abuse* OR use* OR dependan* OR related.ab,ti OR addiction.ab,ti OR addict.ab,ti OR addicts.ab,ti

**4. 1 and 2 and 3**

**Embase (Elsevier: 1974 - November 3, 2021)**

**862 Records**

1. ('family cohesion'/exp OR 'social capital'/exp OR (social NEXT/1 (capital OR cohesion OR control OR organization OR organisation:ab,ti OR 'informal control':ab,ti OR 'collective efficacy':ab,ti OR (family NEAR/2 (cohesion OR organization OR disorganization OR organisation OR disorganisation:ab,ti OR ((community OR neighborhood OR neighbourhood NEAR/2 (cohesion OR participation:ab,ti

2. ('family'/exp OR 'family relation'/exp OR 'family relationships'/exp OR family:ab,ti OR families:ab,ti

3. ('depression'/exp OR 'anxiety disorder'/exp OR 'anger'/exp OR 'hostility'/exp OR 'drug dependence'/exp OR 'drug abuse'/exp OR depression:ab,ti OR depressive:ab,ti OR anxiety:ab,ti OR panic:ab,ti OR obsessive:ab,ti OR ocd:ab,ti OR (stress NEXT/1 (disorder* OR symptom* OR syndrome*:ab,ti OR ((traumatic OR posttraumatic NEXT/1 (stress* OR psycho* OR neurosis:ab,ti OR ptsd:ab,ti OR anger:ab,ti OR rage:ab,ti OR hostility:ab,ti OR alcohol:ab,ti OR alcoholism*:ab,ti OR alcoholic*:ab,ti OR heroin:ab,ti OR opiate*:ab,ti OR opioid*:ab,ti OR opium:ab,ti OR narcotic*:ab,ti OR cannabis:ab,ti OR marijuana:ab,ti OR hashish:ab,ti OR ganja:ab,ti OR cocaine:ab,ti OR methaqualone:ab,ti OR ephedrine:ab,ti OR 'acetic anhydride':ab,ti OR amphetamine*:ab,ti OR inhalant*:ab,ti OR glue:ab,ti OR ((drug* OR substance* NEAR/1 (abuse* OR use* OR dependan* OR related:ab,ti OR addiction:ab,ti OR addict:ab,ti OR addicts:ab,ti

**4. #1 AND #2 AND #3**

**Web of Science (Clarivate Analytics)**

**Indexes=SCI-EXPANDED, SSCI, A&HCI, CPCI-S, CPCI-SSH, BKCI-S, BKCI-SSH, ESCI, CCR-EXPANDED, IC Timespan=All years**

**November 3, 2021**

**1794 Records**

1. TS=(("social" NEAR/1 ("capital" OR "cohesion" OR "control" OR "organization" OR "organisation" OR "informal control" OR "collective efficacy" OR ("family" NEAR/2 ("cohesion" OR "organization" OR "disorganization" OR "organisation" OR "disorganisation" OR (("community" OR "neighborhood" OR "neighbourhood" NEAR/2 ("cohesion" OR "participation"

2. TS=("family" OR "families"

3. TS=("depression" OR "depressive" OR "anxiety" OR "panic" OR "obsessive" OR "ocd" OR ("stress" NEAR/1 ("disorder*" OR "symptom*" OR "syndrome*" OR (("traumatic" OR "posttraumatic" NEAR/1 ("stress*" OR "psycho*" OR "neurosis" OR "ptsd" OR "anger" OR "rage" OR "hostility" OR "alcohol" OR "alcoholism*" OR "alcoholic*" OR "heroin" OR "opiate*" OR "opioid*" OR "opium" OR "narcotic*" OR "cannabis" OR "marijuana" OR "hashish" OR "ganja" OR "cocaine" OR "methaqualone" OR "ephedrine" OR "acetic anhydride" OR "amphetamine*" OR "inhalant*" OR "glue" OR (("drug*" OR "substance*" NEAR/1 ("abuse*" OR "use*" OR "dependan*" OR "related" OR "addiction" OR "addict" OR "addicts"

**4. #1 AND #2 AND #3**

**PsycINFO (EBSCO)**

**November 3, 2021**

**1667 Records**

(DE ("Group Cohesion" OR "Social Capital" OR "Social Control" OR TI ((social N1 (capital OR cohesion OR control OR organization OR organisation OR "informal control" OR "collective efficacy" OR (family W2 (cohesion OR organization OR disorganization OR organisation OR disorganisation OR ((community OR neighborhood OR neighbourhood W2 (cohesion OR participation OR AB ((social N1 (capital OR cohesion OR control OR organization OR organisation OR "informal control" OR "collective efficacy" OR (family W2 (cohesion OR organization OR disorganization OR organisation OR disorganisation OR ((community OR neighborhood OR neighbourhood W2 (cohesion OR participation

AND

(SU (family OR families OR TI (family OR families OR AB (family OR families)

AND

(DE ("Major Depression" OR "Anxiety Disorders" OR "Generalized Anxiety Disorder" OR "Obsessive Compulsive Disorder" OR "Panic Attack" OR "Panic Disorder" OR "Anger" OR "Hostility" OR "Drug Abuse" OR "Inhalant Abuse" OR "Polydrug Abuse" OR "Drug Addiction" OR "Intravenous Drug Usage" OR "Substance Use Disorder" OR "Addiction" OR "Alcohol Use Disorder" OR "Cannabis Use Disorder" OR "Opioid Use Disorder" OR "Intravenous Drug Usage" OR TI (depression OR depressive OR anxiety OR panic OR obsessive OR ocd OR (stress N1 (disorder* OR symptom* OR syndrome* OR ((traumatic OR posttraumatic N1 (stress* OR psycho* OR neurosis OR ptsd OR anger OR rage OR hostility OR alcohol OR alcoholism* OR alcoholic* OR heroin OR opiate* OR opioid* OR opium OR narcotic* OR cannabis OR marijuana OR hashish OR ganja OR cocaine OR methaqualone OR ephedrine OR "acetic anhydride" OR amphetamine* OR inhalant* OR glue OR ((drug* OR substance* N1 (abuse* OR use* OR dependan* OR related OR addiction OR addict OR addicts OR AB (depression OR depressive OR anxiety OR panic OR obsessive OR ocd OR (stress N1 (disorder* OR symptom* OR syndrome* OR ((traumatic OR posttraumatic N1 (stress* OR psycho* OR neurosis OR ptsd OR anger OR rage OR hostility OR alcohol OR alcoholism* OR alcoholic* OR heroin OR opiate* OR opioid* OR opium OR narcotic* OR cannabis OR marijuana OR hashish OR ganja OR cocaine OR methaqualone OR ephedrine OR "acetic anhydride" OR amphetamine* OR inhalant* OR glue OR ((drug* OR substance* N1 (abuse* OR use* OR dependan* OR related OR addiction OR addict OR addicts

**Sociological Abstracts (ProQuest)**

**November 3, 2021**

**929 Records**

(su.exact("Social Cohesion" OR "Social Control" OR ti((social P/1 (capital OR cohesion OR control OR organization OR organisation OR "informal control" OR "collective efficacy" OR (family P/2 (cohesion OR organization OR disorganization OR organisation OR disorganisation OR ((community OR neighborhood OR neighbourhood P/2 (cohesion OR participation OR ab((social P/1 (capital OR cohesion OR control OR organization OR organisation OR "informal control" OR "collective efficacy" OR (family P/2 (cohesion OR organization OR disorganization OR organisation OR disorganisation OR ((community OR neighborhood OR neighbourhood P/2 (cohesion OR participation)

AND

(su(family OR families OR ti(family OR families OR ab(family OR families

AND

(su.exact("Depression (Psychology" OR "Anxiety" OR "Anger" OR "Hostility" OR "Substance Abuse" OR "Drug Abuse" OR "Alcohol Abuse" OR "Addiction" OR "Narcotic Drugs" OR "Alcohol Dependancy" OR ti(depression OR depressive OR anxiety OR panic OR obsessive OR ocd OR (stress N/1 (disorder* OR symptom* OR syndrome* OR ((traumatic OR posttraumatic N/1 (stress* OR psycho* OR neurosis OR ptsd OR anger OR rage OR hostility OR alcohol OR alcoholism* OR alcoholic* OR heroin OR opiate* OR opioid* OR opium OR narcotic* OR cannabis OR marijuana OR hashish OR ganja OR cocaine OR methaqualone OR ephedrine OR "acetic anhydride" OR amphetamine* OR inhalant* OR glue OR ((drug* OR substance* N/1 (abuse* OR use* OR dependan* OR related OR addiction OR addict OR addicts OR ab(depression OR depressive OR anxiety OR panic OR obsessive OR ocd OR (stress N/1 (disorder* OR symptom* OR syndrome* OR ((traumatic OR posttraumatic N/1 (stress* OR psycho* OR neurosis OR ptsd OR anger OR rage OR hostility OR alcohol OR alcoholism* OR alcoholic* OR heroin OR opiate* OR opioid* OR opium OR narcotic* OR cannabis OR marijuana OR hashish OR ganja OR cocaine OR methaqualone OR ephedrine OR "acetic anhydride" OR amphetamine* OR inhalant* OR glue OR ((drug* OR substance* N/1 (abuse* OR use* OR dependan* OR related OR addiction OR addict OR addicts)

**PTSDpubs (ProQuest**

**November 3, 2021**

**227 Records**

(ti((social P/1 (capital OR cohesion OR control OR organization OR organisation OR "informal control" OR "collective efficacy" OR (family P/2 (cohesion OR organization OR disorganization OR organisation OR disorganisation OR ((community OR neighborhood OR neighbourhood P/2 (cohesion OR participation OR ab((social P/1 (capital OR cohesion OR control OR organization OR organisation OR "informal control" OR "collective efficacy" OR (family P/2 (cohesion OR organization OR disorganization OR organisation OR disorganisation OR ((community OR neighborhood OR neighbourhood P/2 (cohesion OR participation)

**Supplementary Material 3**

**Table 2: Search terms for family relationships**

| MEDLINE (Ovid) | Social capital, social cohesion, social control, social organization, informal control. collective efficacy, family cohesion, family organization/disorganization, community/neighborhood cohesion/participation, family/families’ relations/ |
| --- | --- |
| EMBASE (Elsevier) | Family cohesion, social capital, social cohesion, social control, social organization/organization, informal control, collective efficacy, family organization/disorganization, family organization/disorganization, community/neighborhood/neighborhood cohesion/participation, family/families’ relation/relationships |
| Web of Science (Clarivate Analytics) | Social capital, social cohesion, social control, social organization/organization, informal control, collective efficacy, family cohesion, family organization/disorganization, family organization/disorganization, community/neighborhood/neighborhood cohesion/participation, family/families" |
| PycINFO (EBSCO) | Group Cohesion, Social Capital, Social Control, social capital, social cohesion, social control, social organization/organization, informal control, collective efficacy, family cohesion, family organization/disorganization, family organization/disorganization, community/neighborhood/neighborhood cohesion/participation, family/families |
| Sociological Abstracts | Social Cohesion, Social Control, social capital, social cohesion, social control, social organization/organization, informal control, collective efficacy, family cohesion, family organization/disorganization, family organization/disorganization, community/neighborhood/neighborhood cohesion/participation, family/families |
| PTSDPubs | Social capital, social cohesion, social control, social organization/organization, informal control, collective efficacy, family cohesion, family organization/disorganization, family organization/disorganization, community/neighborhood/neighborhood cohesion/participation |

**Supplementary Material 4**

**Figure 1: Flow chart**


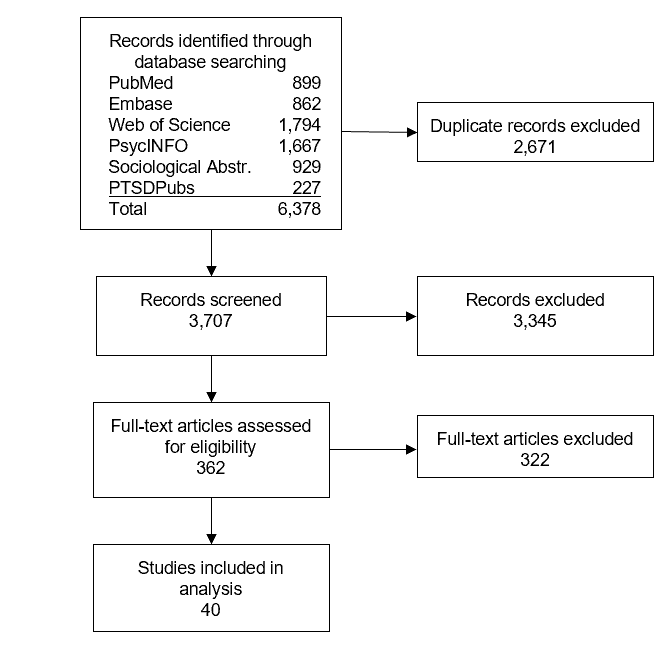


Studies excluded (n = 314)

Age of participants below 18 (n = 82)

Not empirical studies (n = 14)

Duplicates manually excluded (n = 5)

Other topic (n = 10)

Thesis (n = 54)

No full text available (n = 2)

Other outcomes (n = 49)

Other study design (n = 19)

Other exposure (n = 37)

Special Population (n = 42)

**Supplementary Material 5**

**Table 3: Models, summary of model, measurement tools**

| **Model** | **Summary** | **Measurement tool** |
| --- | --- | --- |
| Bowen’s Family System Theory [27] | Differentiation (ability to have active thinking processes during emotional intensity; balance individuality and togetherness), triangulation (bringing a third person into the conflict) | NLAAS measure [28] |
| Circumplex Model of Marital and Family System [18] | Ties in family setting that brings about the recognition of individual family members' rights and respect for such rights, family’s ability to change its power structure, and rules in response to situational and developmental stress, | Family Adaptability and Cohesion Evaluation Scales (FACES) [29] |
|  | belonging (loyalty, fulfilling familial obligations, subjecting one's needs to those of the family, family as a source of security) |  |
| Family expectations [30] | Meeting expectations | Parental Expectations Scale [30] |
| Family functioning [31] | Emotional bonds, belonging/loyalty, reciprocity, harmony in the family | Family Functioning Scale (FFS) [31] |
| Family harmony  [32] | Family atmosphere (getting along vs conflict), responsibility to do housework, time-sharing (sharing vs isolation), seeking help (help seeking vs avoidance), supporting family members (support-providing vs. indifference) | Family Harmony Scale (FHS) [32] |
| Mc Master Model of Family Functioning [33] | Task accomplishment, problem solving, communication, role performance, affective expression, involvement, control, values, and norms | Family Assessment Device (FAD) [33] |
| Psychosocial theory [34] | Family conflict (being close, arguments, loneliness, perceived level of cohesiveness),  satisfaction with communication | Family Communication scale (FCS) [35] |
| Social climate of families [36] | Interpersonal relationship (cohesion, expressiveness, conflict), personal growth (independence), system maintenance | Family Environment Scale (FES) [37] |

**Supplementary material 6**

**Table 4. Study characteristics (authors, country, study design, sample, exposure, outcome, covariates, results) (USA, Italy, Israel, Ghana, Canada, Mexiko, Australia, South Korea, China, Taiwan, 1998 – 2022)**

| **First author** | **Coun-try** | **Study design** | **Sample (n, age^+^ (mean age, SD, age range), sex)** | **Exposure (measure/s)** | **Outcome(s) (measure/s)** | | **Confounder (measure/s)** | **Results*** |
| --- | --- | --- | --- | --- | --- | --- | --- | --- |
| Abe, 2004  [52] | USA | Cross-sectional | N=326, 18-26, Japanese: N=161, mean age: 19.70; Americans: N=165, mean age=20.30, female: n=228 (70.0%), male: n=98 (30.0%) | FC (family cohesion) (FFS: FC Subscale) | Anxiety, depression (STAI-X, BDI) | | Ethnicity, living arrangements, self-esteem (own measure), (RSES) | Japanese: Anxiety: ß=-0.25, p<0.001; depression: ß=-0.22, p<0.001. Americans: Anxiety: ß=0.10, p>0.05; depression: ß=-0.04, p>0.05 |
| Ai et al., 2014 [54] | USA | Cross-sectional | N=1,427, 18-97, mean age=41.14 (SD=16.7), female | Negative family interactions, FC (feelings of closeness) (own measure) | Anxiety, depression, suicidal ideation (WMH-CIDI) | | Acculturation stress, birthplace, discrimination, education, employment, income, English proficiency (own measure, acculturation measure, discrimination measure) | Depression: Negative family interactions: r=0.030, p<0.05; FC: r=-0.019, p>0.05. Anxiety: NFI: r=0.015, p>0.05; FC: r=-0.015, p<0.05; suicidal ideation: NFI: r=0.017, p<0.01; FC: r=-0.015, p<0.01 |
| Ai et al., 2015 [55] | USA | Cross-sectional | N=1,127, 18-92, mean age=39.94 (SD=15.5) | Negative family interactions, FC (own measure) | Anxiety, depression, suicidal ideation (WMH-CIDI) | | Acculturation stress, birthplace, discrimination, education, employment, English proficiency, income (own measure, acculturation measure, discrimination measure) | Negative family interactions: Depression: r=0.0231, p<0.01; FC: r=-0.014, p>0.05. Anxiety: NFI: r=-0.001, p>0.05; FC: r=-0.03, p>0.05. Suicidal ideation: NFI: r=0.008, p>0.05; FC: r=0.001, p>0.05 |
| Bakhtiari et al., 2017 [56] | USA | Cross-sectional | N=164, 18-30, mean age=24.4 (SD=3.8), female: N=87 (53.0%), males: N=77 (47.0%) | Meeting parental expectations, conflicts, PCTS (om, PES) | Depression (CES-D) | Fathers’ birth country, living with parents, marital status, mothers’ birth country, parents’ marital status, self-deprecation (own measure, RSES) | | Unstandardized coefficient FC: -0.18, p<0.05 |
| Bert et al., 2020 [83] | Italy | Cross-sectional | N=2,513, median age: 22, female: N=1,536 (61.3%); male: N=969 (38.7%) | FC | Depression (BDI-II) | Living away from home, nationality, relationship status (om) | | FC: good: OR=0.65 (0.46, 0.93), p=0.019; excellent: OR=0.41, (0.29, 0.59), p<0.001 |
| Bert et al., 2022 [84] | Italy | Cross-sectional | N=203, median age: 20, female: N=166 (81.8%); male: N=37 (n=18.2%) | FC | Depression, suicidal ideation (BDI-II) | Living away from home, nationality, relationship status (om) | | FC: Suicidal ideation: OR=0.18 (0.05, 0.71), p=0.014 |
| Caetano et al., 2017 [57] | USA | Cross-sectional | N=1,510, 18-64, mean age=41.7 (SD=0.36), female: N=815 (54.0%), mals: N=695 (46.0%) | FC (FES, 3 items of FACES) | AUD, Average drinks/week, binge drinking, (own measure, AUD: WMH-CIDI) | Age, gender, education, employment status, income, marital status, religion (om)Attitudes towards drinking, drinking norms | | Low FC: AUD (past 12 months): OR=2.20 (1.21-3.98), p<0.01; binge drinking (past 12 months): OR=1.38 (0.96,1.98), p>0.05; medium FC: AUD: OR=1.88 (1.12, 3.14), p<0.01; binge drinking: OR=0.76 (0.52, 1.12), p>0.05 |
| Caetano, et al., 2018 [58, 59] | USA | Cross-sectional | N=1,510, 18-64, mean age=41.7 (SD=0.36), f: N=815 (54.0%), male: N=695 (46.0%) | FC (FES) 3 items of FACES | Lifetime AUD (WMH-CIDI) | Age, gender, education, attitudes towards drinking, drinking norms, employment, religion information measure, (own measure) | | Low FC: AUD: AOR=2.24 (1.46, 3.44), p<0.001; medium FC: AUD: AOR=2.10 (1.52, 2.91), p<0.001; Low FC: Drug use: OR=2.03 (1.36, 3.02), p<0.001; medium FC: OR=1.50 (1.01, 2.23), p<0.05 |
| Caetano et al., 2019 [60] | USA | Cross-sectional | N=1,510, 18-64, female: N=816 (54.0%), male: N=694 (46.0%) | FC, family pride (FACES III) | AUD (lifetime), drug use (last year), depression (WMH-CIDI, om) | Age, gender, education, employment status, income, marital status, religion (own measure) | | Low FC: Depression: OR=2.13 (1.27, 3.58), p<0.01 |
| Cano et al., 2018 [61] | USA | Cross-sectional | N=411, 18-34, mean age=31.71 (SD=4.99), female: N=189 (46.0%), male: N=222 (54.0%) | FC (FFS) | AUD (AUDIT) | Country of origin, education, household income, partner status, social support (own measure, MOS) | | FC: All: Alcohol use severity: ß=-0.15, p<0.01; ms: ß=-0.33, p<0.01, b=-4.30 (-6.08, -2.52); fs: ß=0.03, p>0.05; b=0.37 (-1.40, 2.13) |
| Cano et al., 2020 [62] | USA | Cross-sectional | N=200, 18-25, mean age: 21.30 (SD=2.09); female: N=102 51.0%); male: N=98 (49.0%) | FC (FC) | Depression (CES-D) | Partner status, nativity, employment, financial strain; resilience; mindfulness; distress tolerance; emotion regulation; social support (own measure; BRS, MAAS, DTS, ERQ, MOS subscale) | | FC: Depression: b=-0.53 (SE=0.58), ß=-0.05, p>0.05 |
| Carris et al., 1998 [63] | USA | Cross-sectional | N=297, 18-20, ma=19.29, (SD=0.74), f: N=168 (56.6%), males: N=129 (43.4%) | Family rigidity (FACES II) | Suicidal ideation (ASIQ) | education, family income, race (own measure, PSI) | | Family rigidity: Chi-Square: 39.40, GFI 0.95 |
| Cheng, 2022  [48] | USA | Longitudinal | N=173, 18-57, mean age=23.05 (SD=7.43), female: N=128 (74.0%), male: N=44 (25.4%), (transgender: N=1 (0.6%) | FC (FACES III) | Depression (PHQ-9) | Years in school, generation, acculturative stress, family cultural conflict (own measure, RASI, HSI) | | FC: Depression t_1_: B=-0.03 (standard error (S)E=0.07), Depression t_2_: B=-0.04 (SE=0.09) (-0.213, 0.137), p>0.05 |
| Darghouth et al., 2015 [64] | USA | Cross-sectional | N=2,554, 18+, female: N=1,428 (55.9%), male: N=1,126 (44.1%) | Family support, - cohesion, - conflict | Distress (K-10) | Age at immigration, country of origin, education, ethnicity, marital status | | Family support: B=-0.01 (-0.01, -0.00), p<0.05;; FC: B=0.01 (-0.00-0.03), p>0.05; Family conflict: B=0.05 (0.04-0.05), p>0.05 |
| Diamond et al., 2008 [82] | Israel | Cross-sectional | N=821, age: 19+, females: N=253 (34.9%), males: N=471 (65.1%) | FC, family adaptability (FACES III) | Drug -, alcohol use (NEQ) | Age, gender, education, profession, attitudes towards drug use (OWN MEASURE, NEQ) | | FC: Alcohol use: χ²=16.40, p<0.001; any drug use: χ²=7.16, p<0.05; cannabis: χ²=8.10, p<0.05; other drugs: χ²=8.10, p<0.05; adaptability: Alcohol use: χ²=8.36, p<0.05, cannabis: χ²=6.31, p<0.05 |
| Dillon et al., 2012 [65] | USA | Cross-sectional | N=527, age range 18-34, mean age=26.95 (SD=4.98), female: N=237 (45.0%), male: N=290 (55.0%) | FC (FFS: FC subscale) | Drug use, alcohol use, harmful alcohol use (TLFB, AUDIT) | Age, gender, marital status, country of origin, immigration status, income prior to immigration, length of stay in the U.S. (own measure) | | FC: Alcohol frequency: IRR=0.87 (0.76, 0.99), p<0.05; alcohol quantity: β=-0.11 (-0.19, -0.02), p<0.01; harmful alcohol use: β=-0.17 (-0.26, -0.08) p<0.001; drug use: OR=0.67 (0.53, 0.84), p< 0.001; frequency of illicit drug use: IRR=1.01 (0.79, 1.27), p>0.05 |
| Escobedo et al., 2018 [66] | USA | Cross-sectional | N=1,445, age range 18-25, ma=23.00 (SD=0.37), females: N=867 (60.0%), males: N=578 (40.0%) | Respect, fidelity, interdependence (Familismo) | Marijuana-, drug use, binge drinking (OM) | Acculturation, fatalism (OM, fatalism measure) | | Difference in 10^th^ & 90^th^ percentile: Familismo: Binge-drinking: 8% (0.7, 15); Respeto: Binge-drinking: -7% (-13, -0.8); marijuana use: -7% (95% CI -12, -1); illicit drug use: -2% (-5, -0.2). |
| Guassi Moreira, 2015 [47] | USA | Longitudinal | N=338, mean age=18.40, (SD=0.36), female: N=217 (64.2%), male: N=121 (35.8%) | FC (Quality, communication, mutual trust) (IPPA) | Depression (CES-D) | Education, family composition, parent’s marital status, residency, optimism, self-esteem (own measure, RSES, LOT-R) | | FC: Depression: B=-2.80, SE=1.01, ß=-0.17, p<0.01 |
| Guo et al., 2015 [67] | USA | Cross-sectional | N=616, age 60+, Asians: N=256, mean age=69.56 (SD=8.25), female: N=141 (55.1%), males: N=115 (44.9%). Latinos: N=360, mean age=69.63 (SD=7.02), female: N=205 (56.91%), males: N=155 (43.09%) | Family relations (cohesion, family support) (FES) | Anxiety disorders, mood disorders (e.g., depression) (WMH-CIDI) | Age, gender, marital status, country of origin, education, English proficiency, family cultural conflict, household income, length of stay in the U.S. (OM) | | Family support: Anxiety: OR=1.95 (1.05, 3.64), p<0.05; OR=1.40 (0.75, 2.62), p>0.05; FC: Anxiety: OR=1.03 (0.64, 1.67), p>0.05. Mood disorders: OR=0.67 (0.46, 0.96), p<0.10 |
| Guo et al., 2018 [68] | USA | Cross-sectional | N=3,158, age range 60-105, mean age=72.81 (SD=8.30), female: N=1,830 (57.95%), males: N=1,328 (42.05%) | Family support, children’s filial piety (Health and Retirement Study, OWN MEASURE) | Anxiety, depression (Anxiety: HADS-A, depression: PHQ-9; PSS-10) | Education, income, marital status, neighborhood cohesion, sense of mastery (OM, ADL, PSS-10) | | Filial piety: Depression: ß=-0.022, p<0.001, family support: ß= 0.182, p<0.01; spouse/partner support: ß=-0.78, p>0.05; Anxiety: Filial piety: ß=-0.024, p<0.001; family support, ß=0.120, p<0.01; partner support ß=0.14, p>0.05 |
| Gyasi et al., 2019 [80] | Ghana | Cross-sectional | N=1,200, age 50+, ma= 66.15 (SD=11.85). male: N=444 (37.0%), female: N=756 (63.0%) | Frequency of family contact (OM) | Psychological distress (K-10) | Educational level, employment status, income, physical activity, social engagement, emotional bonds, self-rated health, functional status, chronic conditions, loneliness (OM, physical activity; GPPAQ, self-health; SF-36, UCLA loneliness scale) | | Family contact: psychological distress: ß=-0.712, SE=0.359, p<0.05 |
| Joel Wong et al., 2012 [69] | USA | Cross-sectional | N=2,072, 18-95, ma=41.20 (SD=14.76), female: N=988 (47.7%), male: N=1,084 (52.3%) | FC (FACES- III: FC subscale) | Suicidal ideation, distress (WMH-CIDI) | English/Asian language proficiency, ethnicity, place of birth, psychological distress (OWN MEASURE, MMCIS-L, K-6) | | Suicidal ideation: OR=0.48 (0.35, 0.66), p<0.001; distress: ß=-0.25, p<0.001 |
| Kwon, 2020  [70] | USA | Cross-sectional | N=1,462, ma=44.59 (SD=13.64), f: N=775 (53.0%), male: N=687 (47.0%) | FC, family conflict, spousal/partner support, spousal/partner strain (FACES IV, subscale of HIS) | Psychological distress (K-10) | Citizenship, discrimination, education, English language proficiency, employment, gender, health status, household income, household size; nativity (Own measure, EDS) | | Family conflict: b=0.230, p<0.001, spousal/partner strain: b=0.064, p<0.01; FC: b=-0.060, p>0.05; spousal/partner support: b=-0.023, p>0.05 |
| Leong et al., 2013 [71] | USA | Cross-sectional | N=4,659, Asians: N=2,095, ma=41.34 (SD=15.57); female: N=1,100 (52.5%), male: N=995 (47.5%), Latinos: n=2,554, mean age=38.02 (SD=15.03), female: N=1,239 (48.5%), males: N=1,315 (51.5%) | FC, Latino: family unity, loyalty, cooperation; Asian: obligation to family responsibilities (FCS) | Anxiety, depression, SUD (Lifetime/12-month prevalence of DSM IV diagnosis) | Acculturative stress, age, discrimination, ethnicity, income, language proficiency (OM, NLAAS measure) | | Latinos: Low FC: anxiety: OR=1.52 (0.37, 6.30), p>0.05; depression: OR=1.11 (0.27, 4.66), p>0.05; SUD: OR=0.08 (0.01, 0.70), p<0.05; Asians: Low FC: anxiety: OR=4.2, depression: OR=1.78 (0.47, 6.81), p>0.05; SUD: OR=1.16 (0.07, 18.43), p>0.05 |
| Levesque & Quesnel-Vallée, 2019  [71] | Canada | Cross-sectional | N=9,890, age range unclear; female: N=5,600, (57.0%); males: N=4,290 (43.0%) | Strength of family ties (own measure) | Heavy episodic drinking (own measure) | Education, income, rural/urban residence, social capital (own measure) | | Family ties: Heavy episodic drinking: males: OR=1.03 (0.76, 1.38), p>0.05, female: OR=0.76 (0.54, 1.09), p>0.05 |
| Litwin & Shiovitz-Ezra, 2011  [72] | USA | Cross-sectional | N=1,462, 65-85, female: N=781 (53.4%), males: N=681 (46.6%) | Family network (OM) | Anxiety (HADS-A) | Education, income, race/ethnicity, religion, comorbidity, (own measure; ADL) | | Family network: anxiety: OR=0.59 (0.36, 0.98), p<0.05 |
| Luna et al., 2020 [50] | Mexico | Cross-sectional | N=161, mean age=19.57 (SD=1.13), female: N=117 (72.7%); males: N=44 (27.3%) | FC, family adaptability (FACES- III) | Anxiety, well-being (BAI, PWBS-A) | Age, gender, marital status, religion, work status, maternal/paternal education (own measure) | | FC separated: OR=2.15 (0.53, 8.60), p=0.27, FC connected: OR=1.13 (0.28, 4.44), p=0.85, FC enmeshed: OR=0.91 (0.22, 3.75), p=0.90; adaptability: structured: OR=0.72 (0.06, 8.75), p=0.79, flexible: OR=0.34 (0.02, 4.38), p=0.40, chaotic: OR=0.54 (0.04, 6.17), p=0.62 |
| Markwick et al., 2015 [52] | Australia | Cross-sectional | Aboriginal subsample: (N=339), age 18+, gender: N/A | Inability to get help from family (own measure) | Mental distress (K-10) | Education, employment status, food security, household income/size, house ownership, marital status, social capital (OM) | | Inability to get help from family: OR=2.31 (1.49, 3.60), p<0.001 |
| Morimoto & Sharma, 2004  [73] | USA | Cross-sectional | N=197, age range: 18-49, mean age=18.90, female: N=146 (74.1%), males: N=51 (25.9%) | FC, parental verbal aggression (PMS, PBI, FACES II, CPTS) | Depression (BDI) | Problem-solving (own measure) | | FC: Depression: ß=-0.29, p<0.01; parental verbal aggression: Depression: ß=0.15, p<0.05 |
| Nam et al., 2016 [85] | South Korea | Cross-sectional | N=302, mean age=40.95 (SD=8.98), female: N=200 (66.2%), males: N=102 (33.8%) | FC, adaptability (FACES III), structure (n family members) | Depression (CES-D) | Education, household composition, household income, migration, traumatic experience, resilience (own measure, CD-RISC) | | FC: Depression: B=-0.041, p<0.001; adaptability: B=0.11, p>0.05; n family members: B=0.92, p>0.05 |
| Park et al., 2014 [67] | USA | Cross-sectional | N=395, 65+, mean age=72.66, (SE=0.38), Asians: N=164, Latinos: N=231, female: N=229 (57.88%), males: N=166 (42.12%) | Family conflict, FC (HSI, FACES-III) | Depression (WMH-CIDI) | Length of stay in the U.S., education, ethnicity, marital status, poverty (own measure) | | All: FC: OR=0.68 (0.54, 0.85), p<0.05; conflict: OR=1.46 (0.96, 2.20), p>0.05. Asians: FC: OR=0.70 (0.38, 1.28), p>0.05; conflict: OR=1.88 (1.71, 3.01), p<0.05. Latinos: FC: OR=0.67 (0.51, 0.87), p<0.05; conflict: OR=1.30 (0.87, 1.97), p>0.05 |
| Park, 2017 [49] | South Korea | Longitudinal | N=2,435, 65-104 | Family size (own measure) | Depression (CES-D) | Education, marital status, poverty (own measure) | | Family size: ß=0.407, p<0.001 |
| Priest & Denton, 2012  [75] | USA | Cross-sectional | N=2,554, age 18+, gender: N/A | FC, family conflict (FACES III) | Anxiety disorders; PTSD (WMH-CIDI) | Age, gender, education, income, Acculturation, ethnicity, marital status (own measure) | | FC: anxiety: B=-0.05, OR=0.947 (0.901, 0.994), p<0.05; PTSD: B=-0.03, OR=0.971 (0.931, 1.012), p>0.05; conflict: anxiety: B=0.17, OR=1.180 (1.041, 1.339), p<0.05, PTSD: B=0.19, OR=1.213 (1.033, 1.425), p<0.05 |
| Rivera et al., 2008 [76] | USA | Cross-sectional | N=2,540, age 18+, gender: N/A | FC (FACES- III) | Distress (K-10) | Education, ethnicity, family cultural conflict, income, marital status, migration history (own measure) | | FC: B=-0.43 (SE=0.19), p<0.05 |
| Savage & Mezuk, 2014  [77] | USA | Cross-sectional | N=4,649, age: 18+; Asian N=2,095, Latino N=2,554; female: N=2,524 (54.3%), males: N=2,125 (45.7%) | FC, family conflict (FACES III) | AUD, DUD (WMH-CIDI, GAD) | Age, gender, socioeconomic status, marital status, geographic region, acculturation, neighborhood characteristics, discrimination (own measure) | | FC: AUD/DUD; OR=0.90 (0.77, 1.04), p=0.16; family conflict: OR=1.02 (0.91, 1.13), p=0.79 |
| Wang et al., 2021 [78] | USA | Cross-sectional | N=1,439, mean age=66.72 (SD=8.58), female: N=896 (55.42%), males: N=543 (44.58%) | Negative family interactions (own measure) | Depression: (CES-D; WMH-CIDI) | Chronic health conditions, education, employment, family income, home ownership, marital status, nativity of household residents, region, race (own measure) | | Negative family interactions: Depression: B=1.91 (SE=0.35), p<0.001 |
| Westrick et al., 2021 [79] | USA | Cross-sectional | N=517, mean age=34.92 (SD=4.86), female: N=237 (45.8%), males: N=280 (54.2%) | Family support, FC (FFS) | AUDIT | Annual household income, cultural identification /practices, education, family history of alcohol use, marital status, region of origin (own measure; BIQ; MEIM) | | FC: Alcohol use: ß= -0.49 (-0.82, -0.17), p=0.003 |
| Xie et al., 2021 [81] | China | Cross-sectional | N=689, mean age=29.03 (SD=4.9), female | FC, family conflict (FES) | Anxiety, depression (SCL-90) | Maternal age, gestational age, sleep quality (own measure, PSQI) | | FC: Anxiety: r=-0.303, p<0.001, depression: r=-0.413, p<0.001, family conflict: Anxiety: r=0.024, p<0.001, depression: r=0.307, p<0.001 |
| Yang et al., 2014 [86] | Taiwan | Cross-sectional | N=268, age: N/A, female | Family adaptability, FC (FACES III) | Depression (BSRS-5) | Language that mother uses, mother’s education/occupation, original nationality, discrimination | | Family adaptability: Depression: ß=0.60 (SE=0.36), p>0.05, FC: ß=0.70 (SE=0.43), p>0.05 |

**Abbreviations:**

**Outcomes:** AUD: Alcohol Use Disorder; SUD: Substance Use Disorder

**Measures outcomes**: ADL: The Activities of Daily Living Questionnaire; ASIQ: Adult Suicidal Ideation Questionnaire; AUDIT: Alcohol Use Disorders Identification Test; BAI: Beck Anxiety Inventory; BDI: Beck Depression Inventory; BDI-II: Beck Depression Inventory-II; BIQ: Bicultural Involvement Questionnaire; BRS: Brief Resilience Scale; BSRS: Brief Symptom Rating Scale; CD-RISC: Connor-Davidson Resilience Scale; CES-D: The Center for Epidemiological Studies-Depression; CIDI: Composite International Diagnostic Interview; CTS: The Conflict Tactics Scale; DTS: Distress Tolerance Scale; EDS: Everyday Discrimination Scale; FACES II: Family Adaptability and Cohesion Scale-II; FACES III: Family Adaptability and Cohesion Scale-III; FACES IV: Family Adaptability and Cohesion Scale-IV; FES: Family Environment Scale; FFS: Family Functioning Scale; GPPAQ: General Practice Physical Activity Questionnaire; HADS-A: Hospital Anxiety and Depression Scale-Anxiety; HSI: Family cultural conflict subscale of the Hispanic Stress Inventory; IIP: The Inventory of Interpersonal Problems; IPPA: Inventory of Parent and Peer Attachment; K-6: Kessler Psychological Distress Scale; K-10: Kessler Psychological Distress Scale; LOT-R: Life Orientation Test Revised; MAAS: Mindful Attention Awareness Scale; MMCIS-L: Multidimensional Measure of Cultural Identity Scales for Latinos; MEIM: Multi-Group Ethnic Identity Measure; MOS: Instrument of the Medical Outcomes Study Social Support Survey; NEQ: National Epidemiological Questionnaire; PBI: The Parental Bonding Instrument; PHQ-9: Patient Health Questionnaire; PMS: The Psychological Maltreatment Scale; PSQI: Pittsburgh Sleep Quality Index; PSI: Problem Solving Inventory; PSS-10: Perceived Stress Scale; PWBS-A: Psychological Well-being Scale for Adults; RASI: Riverside Acculturation Stress Inventory; RSES: Rosenberg Self-Esteem Scale; SCL-90: The Symptom Checklist 90; SF-36: The Short Form (36) Health Survey; STAI-X: State-Trait Anxiety Inventory; TLFB: Timeline Follow-back Interview, WMH-CIDI: World Mental Health Survey Initiative Version

**Supplementary Material 7**

**Table 5: Mental health conditions, measures to assess mental health conditions and studies included in the systematic review**

| **Mental health conditions** | **Measures (acronym)** | **First author, year** |
| --- | --- | --- |
| **Anxiety** | Beck Anxiety Inventory (BAI) | Luna et al., 2020 (50) |
|  | World Mental Health Composite Diagnostic Interview (WMH-CIDI) | Ai et al., 2014 [54]; Ai et al., 2015 [55]; Guo et al., 2015 [67]; Priest et al., 2012 [75] |
|  | Hospital Anxiety and Depression Scale (HADS-A) | Guo et al., 2018 [68]; Litwin et al., 2011 [72] |
|  | Symptom Checklist 90 (SCL-90) | Xie et al., 2021 [81] |
|  | State-Trait- Anxiety Inventory (STAI-X) | Abe et al., 2004 [52] |
| **Alcohol abuse** | Alcohol Use Disorders Identification Test (AUDIT) | Cano et al., 2018 [61]; Dillon et al., 2012 [65]; Westrick et al., 2021 |
|  | Own measure | Caetano et al., 2017 [57]; 2018 [59], Escobedo et al. ,2018 [66]; Levesque et al., 2019 [51] |
|  | National Epidemiological Questionnaire (NEQ) | Diamond et al., 2008 [82] |
|  | World Mental Health Composite Diagnostic Interview (WMH-CIDI) | Caetano et al., 2017 [57]; 2018 [59]; 2019 [60]; Savage et al. 2014 [77] |
| **Depression** | Beck Depression Inventory (BDI) | Abe et al., 2004 [52]; Morimoto et al., 2004 [73] |
|  | Beck Depression Inventory II (BDI-II) | Bert et al., 2020 [83]; Bert et al., 2022 [84] |
|  | Brief Symptom Rating Scale (BSRS-5) | Yang et al., 2014 [86] |
|  | Center for Epidemiologic Studies Depression Scale (CES-D) | Bakhtiari et al., 2017 [56]; Cano et al., 2020 [62]; Guassi Moreira et al., 2015 [47]; Nam et al., 2016 [85]; Park et al., 2017 [49]; Wang et al., 2021 [78] |
|  | Composite International Diagnostic Interview (CIDI) | Ai et al., 2014 [54]; 2015 [55]; Caetano et al., 2019 [60]; Park et al., 2014 [74]; Wang et al., 2021 [78]; Joel Wong et al., 2012 [69] |
|  | DSM-IV diagnosis | Leong et al., 2013 [71] |
|  | Patient Health Questionnaire 9 (PHQ-9) | Cheng et al., 2022 [48]; Guo et al., 2018 [68] |
|  | Symptom Checklist-90 (SCL-90) | Xie et al., 2021 [81] |
| **Distress** | Kessler Psychological Distress Scale (K-10) | Darghouth et al., 2015 [64]; Gyasi et al., 2019 [80]; Kwon et al., 2020 [70]; Markwick et al., 2015 [53]; Rivera et al., 2008 [76] |
| **Drug use** | DSM-IV diagnosis | Leong et al., 2013 [71] |
|  | World Mental Health Composite Diagnostic Interview (WMH-CIDI) | Savage et al., 2014 [77] |
|  | National Epidemiological Questionnaire (NEQ) | Diamond et al., 2008 [82] |
|  | Own measure | Caetano et al., 2018b [59]; 2019 [60]; Escobedo et al., 2018 [66] |
|  | Timeline Follow-Back Interview (TFLB) | Dillon et al., 2012 [65] |
| **Mood disorders** | World Mental Health Composite Diagnostic Interview (WMH-CIDI) | Guo et al., 2015 [67] |
| **PTSD** | World Mental Health Composite Diagnostic Interview (WMH-CIDI) | Priest et al., 2012 [75] |
| **Suicidal ideation** | Adult Suicidal Ideation Questionnaire (ASIQ) | Carris et al., 1998 [63] |
|  | Beck Depression Inventory II (BDI-II) | Bert et al., 2022a [83] |
|  | World Mental Health Composite Diagnostic Interview (WMH-CIDI) | Joel Wong et al., 2012 [69] |

**Supplementary Material 8**

**Table 6: Risk of Bias of cross-sectional studies in the systematic review on family relationships and mental health outcomes**

| **Author,**  **year** | **1**  **Is the research question clearly focused?** | **2**  **Is the study design appropriate?** | **3**  **Clear description of election/recruitment of participants?** | **4**  **Is there a selection bias?** | **5**  **Is the sample representative of the population?** | **6**  **Is the sample size based on power calculation?** | **7**  **Is the response rate satisfactory?** | **8**  **Use of valid measurements (questionnaires?** | **9**  **Use of reliable measurements?** | **10**  **Assessment of statistical significance?** | **11**  **Were confidence intervals given?** | **12**  **Are there confounding factors that have not been accounted for?** |
| --- | --- | --- | --- | --- | --- | --- | --- | --- | --- | --- | --- | --- |
| Abe et al., 2004 [52] | Y | Y | Y | N | Y | N | Y | Y | Y | Y | N | Y |
| Bakhtiari et al., 2017 [56] |  |  |  |  |  |  |  |  | Y |  |  | Y |
|  | Y | Y | Y | N | Y | N | Y | Y | Y | Y | Y | Y |
| Bert et al. 2022 [84] | Y | Y | Y | N | Y | N | U | Y | Y | Y | Y | N |
|  |  |  |  |  |  |  |  |  |  |  |  |  |
| Bert et al., 2020 [83] | Y | Y | Y | N | Y | Y | Y | Y | Y | Y | Y | N |
|  |  |  |  |  |  |  |  |  |  |  |  |  |
| Caetano et al., 2017 [57] | Y | Y | Y | N | Y | N | Y | Y | Y | Y | Y | Y |
|  |  |  |  |  |  |  |  |  |  |  |  |  |
| Caetano et al., 2018a [58] | Y | Y | Y | N | Y | N | Y | Y | Y | Y | Y | Y |
|  |  |  |  |  |  |  |  |  |  |  |  |  |
| Caetano et al., 2018b [58] | Y | Y | Y | N | Y | N | Y | Y | Y | Y | Y | Y |
|  |  |  |  |  |  |  |  |  |  |  |  | Y |
| Caetano et al., 2019 [60] | Y | Y | Y | N | Y | N | Y | Y | Y | Y | Y | Y |
|  |  |  |  |  |  |  |  |  |  |  |  |  |
| Cano et al., 2018 [61] | Y | Y | Y | Y | Y | N | Y | Y | Y | Y | Y | N |
|  |  |  |  |  |  |  |  |  |  |  |  |  |
| Cano et al. [2020] | Y | Y | Y | Y | Y | N | U | Y | Y | Y | N | N |
|  |  |  |  |  |  |  |  |  |  |  |  |  |
| Carris et al. [1998] | Y | Y | N | U | Y | N | Y | Y | Y | Y | N | Y |
|  |  |  |  |  |  |  |  |  |  |  |  |  |
| Darghouth et al. [2015] | Y | Y | Y | N | Y | Y | Y | Y | Y | Y | Y | N |
|  |  |  |  |  |  |  |  |  |  |  |  |  |
| Diamond et al., 2008 [82] | Y | Y | N | N | Y | N | Y | Y | Y | Y | N | Y |
|  |  |  |  |  |  |  |  |  |  |  |  |  |
| Dillon et al., 2012 [65] | Y | Y | Y | N | N | U | U | Y | Y | Y | Y | N |
|  |  |  |  |  |  |  |  |  |  |  |  |  |
| Escobedo et al., 2018 [66] | Y | Y | Y | N | Y | N | U | N | N | Y | Y | Y |
|  |  |  |  |  |  |  |  |  |  |  |  |  |
| Guo et al., 2015 [67] | Y | Y | Y | N | Y | Y | Y | Y | Y | Y | Y | N |
|  |  |  |  |  |  |  |  |  |  |  |  |  |
| Guo et al., 2018 [68] | Y | Y | Y | N | Y | U | Y | Y | Y | Y | N | N |
|  |  |  |  |  |  |  |  |  |  |  |  |  |
| Gyasi et al., 2019 [80] | Y | Y | Y | N | Y | Y | Y | Y | Y | Y | N | N |
|  |  |  |  |  |  |  |  |  |  |  |  |  |
| Joel Wong et al., 2012 [69] | Y | Y | Y | N | Y | Y | Y | Y | Y | Y | Y | N |
|  |  |  |  |  |  |  |  |  |  |  |  |  |
| Kwon, 2020 [70] | Y | Y | Y | N | Y | Y | Y | Y | Y | Y | N | N |
|  |  |  |  |  |  |  |  |  |  |  |  |  |
| Leong et al., 2013 [71] | Y | Y | Y | N | Y | Y | Y | Y | Y | Y | Y | N |
|  |  |  |  |  |  |  |  |  |  |  |  |  |
| Levesque et al., 2019 [51] | Y | Y | N | U | N | N | U | N | N | Y | Y | Y |
|  |  |  |  |  |  |  |  |  |  |  |  |  |
| Litwin et al., 2011 [72] | Y | Y | Y | N | Y | N | Y | Y | Y | Y | Y | N |
|  |  |  |  |  |  |  |  |  |  |  |  |  |
| Luna et al., 2020 [50] | Y | Y | N | N | U | N | Y | Y | Y | Y | N | N |
|  |  |  |  |  |  |  |  |  |  |  |  |  |
| Markwick et al., 2015 [53] |  |  |  |  |  |  |  |  |  |  |  |  |
|  | Y | Y | Y | N | Y | Y | Y | Y | Y | Y | Y | N |
| Morimoto et al., 2004 [73] | Y | Y | Y | Y | Y | N | Y | Y | Y | Y | N | Y |
|  |  |  |  |  |  |  |  |  |  |  |  |  |
| Nam et al., 2016 [85] | Y | Y | Y | N | Y | N | Y | Y | Y | Y | Y | N |
|  |  |  |  |  |  |  |  |  |  | Y |  |  |
| Park et al., 2014 [74] | Y | Y | Y | N | Y | Y | Y | Y | Y | Y | Y | N |
|  |  |  |  |  |  |  |  |  |  |  |  | N |
| Priest et al., 2012 [75] | Y | Y | Y | N | Y | Y | Y | Y | Y | Y | Y | N |
|  |  |  |  |  |  |  |  |  |  |  |  |  |
|  | Y | Y | Y | N | Y | Y | Y | Y | Y | Y | N | N |
| Savage Jet al., 2014 [77] |  |  |  |  |  |  |  |  |  |  |  |  |
|  | Y | Y | Y | N | Y | Y | Y | Y | Y | Y | Y | N |
| Wang et al., 2021 [78] | Y | Y | Y | N | Y | U | Y | Y | Y | Y | Y | N |
|  |  |  |  |  |  |  |  |  |  |  |  |  |
| Westrick et al., 2021 [79] | Y | Y | Y | U | U | N | U | Y | Y | Y | Y | N |
|  |  |  |  |  |  |  |  |  |  |  |  |  |
| Xie et al., 2021 [81] | Y | Y | Y | N | N | N | U | Y | Y | Y | N | Y |
|  |  |  |  |  |  |  |  |  |  |  |  |  |
| Yang et al., 2014 [86] | Y | Y | Y | N | Y | N | Y | Y | Y | Y | N | Y |
|  |  |  |  |  |  |  |  |  |  |  |  |  |

**Supplementary Material 9**

**Table 7: Risk of Bias of cohort studies in the systematic review on family relationships and mental health outcomes**

| **Author, Year** | **1**  **Is the research question clearly focused?** | **2**  **Are the participants recruited in an acceptable way?** | **3**  **Is the exposure accurately measured to minimise bias?** | **4**  **Is the outcome accurately measured to minimise bias?** | **5**  **Are all important confounding factors identified?** | **6**  **Are the confounding factors taken into account in the design and/or analysis?** | **7**  **Is the follow-up complete enough?** | **8**  **Is the follow-up long enough?** | **9**  **Do you believe the results?** | **10**  **Can the results be applied to the local population?** | **11**  **Do the results fit with other evidence?** |
| --- | --- | --- | --- | --- | --- | --- | --- | --- | --- | --- | --- |
| Cheng, 2022 [40] | Y | Y | Y | Y | N | Y | N | Y | Y | N | Y |
|  |  |  |  |  |  |  |  |  |  |  |  |
| Guassi Moreira et al., 2015 [47] | Y | Y | Y | Y | N | Y | Y | N | Y | U | Y |
|  |  |  |  |  |  |  |  |  |  |  |  |
| Park et al., 2017  [42] | Y | Y | N | Y | Y | N | Y | Y | Y | N | Y |
|  | Y | Y | N | Y | Y | N | Y | Y | Y | N | Y |

**Supplementary Material 10**

**Figure 2: Funnel plot for studies on positive family relationships and depression**


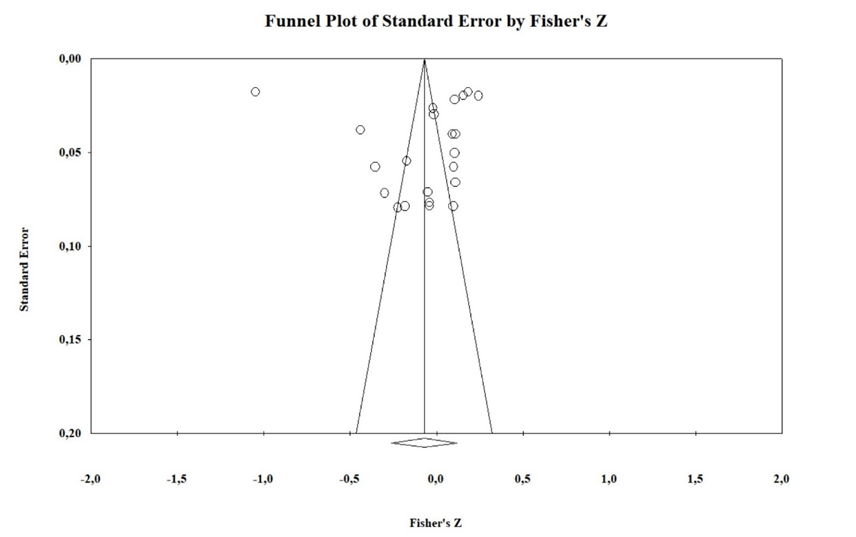


**Supplementary Material 11**

**Figure 3: Funnel plot of the random effects model for negative family relationships and depression**


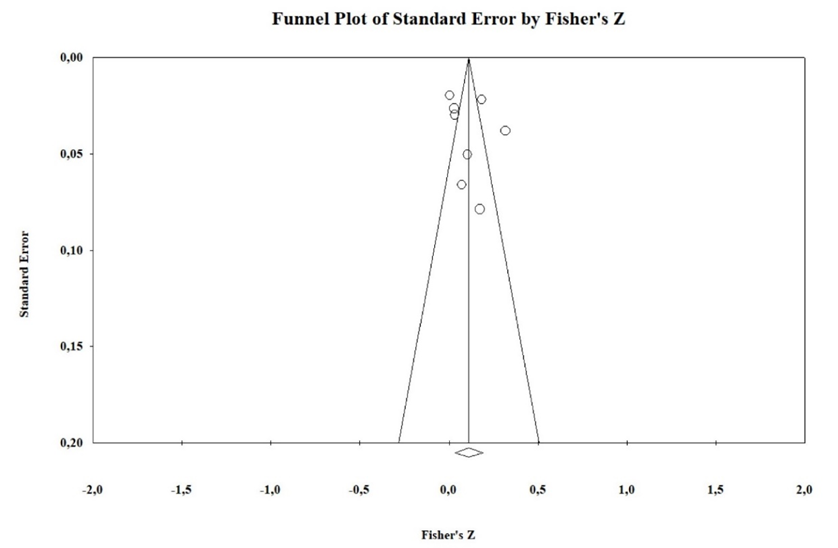


**Supplementary Material 12**

**Figure 4. Funnel plot for studies on positive family relationships and anxiety**


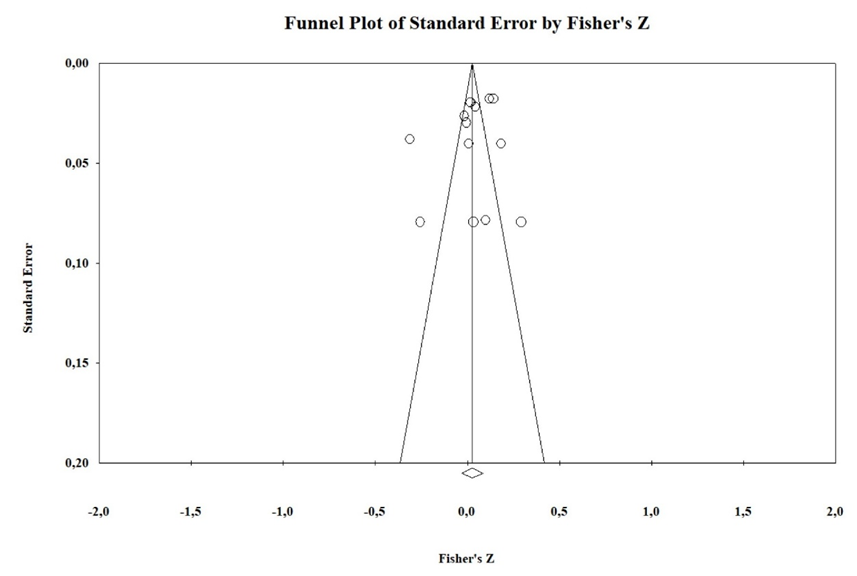


**Supplementary Material 13**

**Figure 5: Funnel plot for studies on negative family relationships and anxiety**


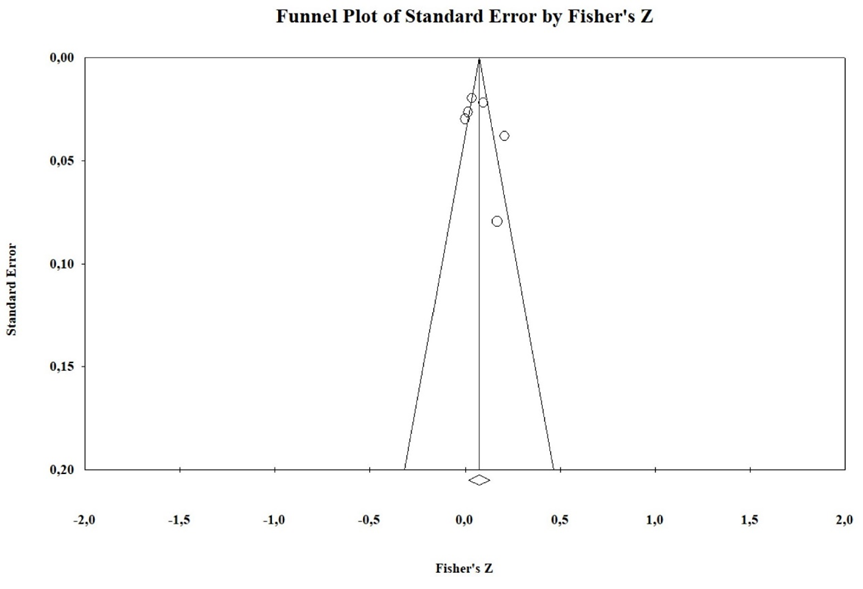


**Supplementary Material 14**

**Figure 6: Funnel plot for studies on positive family relationships and alcohol abuse**


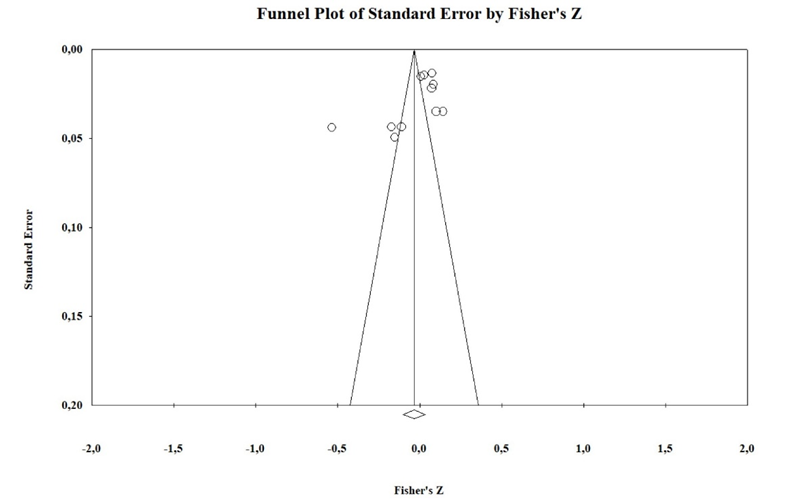


**Supplementary Material 15**

**Figure 7: Funnel plot for studies on positive family relationships and illicit substances use**


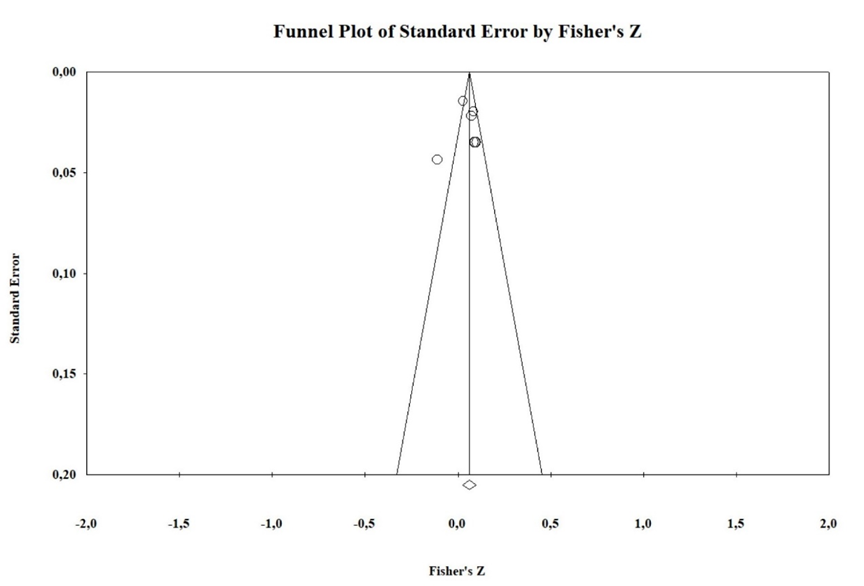


**Supplementary Material 16**

**Figure 8: Associations between positive family relationships and alcohol abuse (Worldwide, 2008 – 2021)**


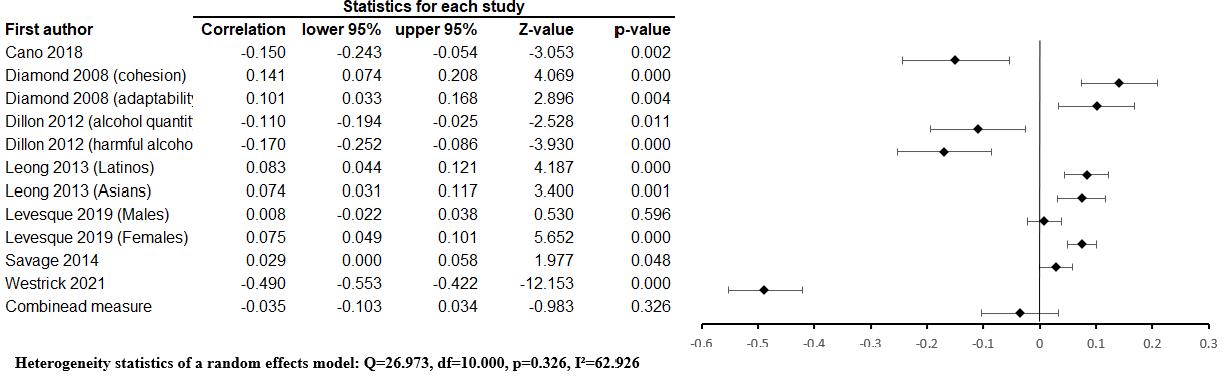


**Supplementary Material 17**

**Figure 9: Associations between positive family relationships and use of illicit substances (Worldwide, 2008 – 2014)**


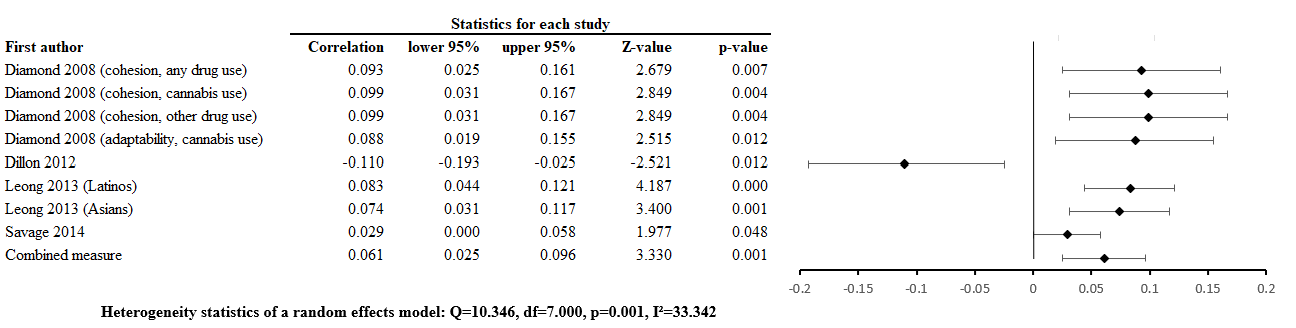


**Supplemental Material 18**

**References**

[1] World Health Organization (WHO). Depression and Other Common Mental Disorders Global Health Estimates (2017). https://apps.who.int/iris/handle/10665/254610 [Accessed April 8^th^, 2024].

[2] Global Burden of Disease Mental Disorders Collaborators. Global, regional, and national burden of 12 mental disorders in 204 countries and territories, 1990-2019: a systematic analysis for the Global Burden of Disease Study. Lancet Psychiatry (2019) 9(2):137-150. doi: 10.1016/ S2215-0366(21)00395-3

[3] Fekadu W, Mihiretu A, Craig TKJ, Fekadu A. Multidimensional impact of severe mental illness on family members: systematic review. *BMJ Open* (2019) 9:e032391. doi: 10.1136/bmjopen-2019-032391

[4] Arias D, Saxena S, Verguet S. Quantifying the global burden of mental disorders and their economic value. *EClinicalMedicine* (2022) 54:101675. doi: 10.1016/j.eclinm.2022.101675

[5] Woods SB. Biopsychosocial theories. In: Fiese BH. Deater-Deckard K, Jouriles EN. Whisman M (editor) APA Handbook of Contemporary Family Psychology. Washington DC: American Psychological Association (2019).

[6] McDermott BM, Batik M, Roberts L, Gibbon P. Parent and child report of family functioning in a clinical child and adolescent eating disorders sample. Aust NZ J Psychiatry (2022) 36(4):509-14. doi: 10.1046/j.1440-1614.2002.01043.x

# [7] Lum TTY, Yan ECW, Ho AHY, Shum MHY, Wong GHY, Lau MMY et al. Measuring Filial Piety in the 21st Century: Development, Factor Structure, and Reliability of the 10-Item Contemporary Filial Piety Scale. J Appl Gerontol (2016) 35,11:1235-47. doi: 10.1177/0733464815570664

[8] Axia VD, Weisner TS. Infant stress reactivity and Home Cultural Ecology of Italian infants and families. *Infant Behav Dev* (2002) 25:255-268. doi: 10.1016/S0163-6383(02)00099-1

[9] Aquilino WS, Supple AJ. Long-term effects of parenting practices during adolescence on well-being: Outcomes in young adulthood. *J Fam Issues* (2001) 22(3):289-308. doi: 10.1177/019251301022003002

[10] Baltes PB. Theoretical propositions of lifespan developmental psychology: On the dynamics between growth and decline. Dev Psychol (1987) 23(5):611-26. doi: 10.1037/0012-1649.23.5.611

[11] Carr A. The Evolution of Systems Theory. In Sexton TL, Lebow J. (Eds.) Handbook of family therapy. New York, NY: Routledge/Taylor & Francis Group (2015). p. 13-29.

[12] Fiese BH, Celano M. Deater-Deckard K, Jouriles EN, Whisman MA (Eds.). APA Handbook of contemporary family psychology: Foundations, methods, and contemporary issues across the lifespan. Washington, DC: American Psychological Association (2019).

[13] Bronfenbrenner U, Morris, PA. The bioecological model of human development. In W. Damon (Series Ed.) RM. Lerner (Vol. Ed.) Handbook of child psychology; Vol. 1: Theoretical models of human development. New York: Wiley (2006). p. 793-828.

[14] Conger RD, Wallace LE, Sun Y, Simons RL, McLoyd VC. Economic pressure in African American families: A replication and extension of the family stress model. Dev Psychol (2002) 38(2):179-93. doi: 10.1037/0012-1649.38.2.179

[15] Lerner RM. Handbook of child psychology: Theoretical models of human development, Vol. 1, 6th ed. Lerner, RM, Damon W. editors. Hoboken, NJ, US: John Wiley & Sons Inc (2006).

[16] Bowlby J. Attachment and Loss. Vol. 3: Loss, Sadness, and Depression. New York: Basic Books. (1980).

[17] Bowlby J. Attachment and loss: retrospect and prospect. *Am J Orthopsychiat* (1982) 52(4):664-678. doi: 10.1111/j.1939-0025.1982.tb01456.x

[18] Olson DH. Circumplex Model of Marital and Family Systems. *J Fam Ther* (2008) 22(2):144–67. doi: 10.1111/1467-6427.00144

[19] Cardamone-Breen MC, Jorm AF, Lawrence KAMA, Yap MBH. The Parenting to Reduce Adolescent Depression and Anxiety Scale: Assessing parental concordance with parenting guidelines for the prevention of adolescent depression and anxiety disorders. Peer J (2017) 5:e3825. doi: 10.7717/peerj.3825

[20] Coleman J (1988) Social capital in the creation of human capital. American Journal of Sociology, 1988, 94: 95-120

[21] Amato P. More than money? Men`s contribution to their children`s lives. In: Booth A, Creuter A (eds) Men in families: When do they get involved? What difference does it make? Lawrence Erlbaum, New Jersey 1998.

[22] Furstenberg F. Social capital and successful development among at risk youth. Journal of Marriage and the family, 1995, 57: 580-92.

[23] Bourdieu P. Sociology in question. Sage, London 1993.

[24] Putnam RD. Bowling Alone: The Collapse and revival of American Community. New York: Touchstone 2001.

[25] Fukuyama F. The great disruption: Human nature and the reconstruction of social order. Free Press, New York 1999.

[26] Schrodt P, Witt PL, Messersmith AS. A meta-analytical review of family communication patterns and their associations with information processing, behavioral, and psychosocial outcomes. Comm Mono (2008) 75:248-69. doi: 10.1080/03637750802256318

[27] Bowen M. Family therapy in clinical practice. Northvale, NJ: Jason Aronson Inc. (1978).

[28] Alegria M, Vila D, Woo M, Canino G, Takeuchi D, Vera M, et al. Cultural relevance and equivalence in the NLAAS Instrument: Integrating etic and emic in the development of cross-cultural measures for a psychiatric epidemiology and services study of Latinos. Inter J Meth Psych Res (2004) 13(4):270-88. doi: 10.1002/mpr.181

[29] Olson D. FACES IV and the circumplex model: A validation study. J Marital Fam Ther (2011) 37(1):64-80. doi: 10.1111/j.1752-0606.2009.00175.x

[30] Sasikala S, Karunanidhi S. Development and validation of perception of parental Expectations Inventory. J Indian Acad Appl Psychol (2011) 37(1):114-124.

[31] Tavitian ML, Lubiner J, Green L, Grebstein LC, Velicer WF. Dimensions of family functioning. J Soc Behav Pers (1987) 2:191-204.

[32] Conger RD, Conger KJ, Martin MJ. Socioeconomic status, family processes, and individual development. J Marriage Fam (2010) 72:685-704. doi: 10.1111/j.1741-3737.2010.00725.x

[33] Kavikondala S, Stewart SM, Ni MY, Chan BHY, Lee PH, Li K-K, et al. Structure and validity of Family Harmony Scale: An instrument for measuring harmony. *Psychol Assessment* (2016) 28(3):307-18. doi: 10.1037/pas0000131

[34] Epstein NB, Baldwin LM, Bishop DS. The McMaster family assessment device. *J marital fam ther* (1983) 9(2):171-80. doi: 10.1111/j.1752-0606.1983.tb01497.x

[35] Billings AC, Moos RH. Psychosocial theory and research on depression: An integrative framework and review. Clin Psychol Rev 2 (1982):213-237. doi: [10.1016/0272-7358(82)90013-7](https://doi.org/10.1016/0272-7358(82)90013-7)

[36] Caughlin JP. Family Communication Standards. Hum Commun Res (2003) 29:5-40. doi: 10.1111/j.1468-2958.2003.tb00830.x

[30] Lazarus RS, Folkman S. Transactional theory and research on emotions and coping. Eur J Personality 1 (1987):141-169. doi: 10.1002/per.2410010304

[31] Moos RH, Moos BS. Family Environment Scale Manual: Development, Applications, Research - Third Edition. Palo Alto, CA: Consulting Psychologist Press (1994).

[37] Borenstein M, Hedges LV, Higgins JPT, Rothstein HR. Introduction to Meta‐Analysis. John Wiley & Sons, Ltd. (2009).

[38] Corey DM, Dunlap WP, Burke M. Averaging correlations: Expected values and bias in combined Pearson r's and Fisher's z transformations. J Gen Psychol (1998) 125:245-62. doi: 10.1080/00221309809595548

[39] Silver NC, Dunlap WP. Averaging correlation coefficients: Should Fisher's z transformation be used? *J Appl Psychol* (1987) 72:146–48. doi: 10.1037/0021-9010.72.1.146

[40] Hedges LV, Vevea JL. Fixed- and random-effects models in meta-analysis. Psychol Methods (1998) 3:486-504. doi: 10.1037/1082-989X.3.4.486

[41] Marín-Martínez F, Sánchez-Meca J. Weighting by inverse variance or by sample size in random-effects meta-analysis. Edu psychol measure (2010) 70(1):56-73. doi: 10.1177/0013164409344534

[42] Cohen J. Statistical power analysis for the behavioral sciences (2nd ed.) Hillsdale, New Jersey: Lawrence Erlbaum Associates; Inc. (1988).

[43] Huedo-Medina TB, Sánchez-Meca J, Marín-Martínez F, Botella J. Assessing heterogeneity in meta-analysis: Q statistic or I^2^ index? *Psychol Methods* (2006) 11(2):193-206. doi: 10.1037/1082-989X.11.2.193

[44] Higgins JP, Thompson SG. Quantifying heterogeneity in a meta-analysis. *Stat Med*. (2002) 21(11):1539-1558. doi: 10.1002/sim.1186

[45] Begg CB, Mazumdar M. Operating characteristics of a rank correlation test for publication bias. *Biometrics*. (1994) 50(4):1088-1101. doi: 10.2307/2533446

[46] Egger M, Davey Smith G, Schneider M, Minder C. Bias in meta-analysis detected by a simple, graphical test. *BMJ* (1997) 315(7109):629-634. doi: 10.1136/bmj.315.7109.629

[47] Guassi Moreira JF, Telzer EH. Changes in family cohesion and links to depression during the college transition. J Adolesc (2015) 43:72-82. doi: 10.1016/j.adolescence.2015.05.012

[48] Cheng H-L. Acculturative stress, family relations, and depressive symptoms among Latinx college students: A cross-lagged study. J Latinx Psychol (2022) 10(1):39-53. doi: 10.1037/lat0000197

[49] Park MJ. Impact of social capital on depression trajectories of older women in Korea. J Aging Ment Health (2017) 21(4):354-361. doi: 10.1080/13607863.2015.1088511

[50] Luna D, Urquiza-Flores DI, Figuerola-Escoto RP, Carreño-Morales C, Meneses-González F. Academic and sociodemographic predictors of anxiety and psychological well-being in Mexican medical students. A cross-sectional study. Predictores académicos y sociodemográficos de ansiedad y bienestar psicológico en estudiantes mexicanos de medicina. Estudio transversal. *Gac Med Mex* (2020) 156(1):40-46. doi: 10.24875/GMM.19005143

[51] Levesque A, Quesnel-Vallée A. Gender variations in the relationship between social capital and mental health outcomes among the Indigenous populations of Canada. Int J Equity Health (2019) 18(1):124. doi: 10.1186/s12939-019-1028-9

[52] Abe JAA. Self-esteem, perception of relationships, and emotional distress: A cross-cultural study. *Pers Relationship* (2004) 11(2):231–47. doi: 10.1111/j.1475-6811.2004.00080.x

[53] Markwick A, Ansari Z, Sullivan M, McNeil J. Social determinants and psychological distress among Aboriginal and Torres Strait islander adults in the Australian state of Victoria: a cross-sectional population based study. *Soc Sci Med* (2015) 128:178-187. doi: 10.1016/j.socscimed.2015.01.014

[54] Ai AL, Weiss SI, Fincham FD. Family factors contribute to general anxiety disorder and suicidal ideation among Latina Americans. *Women`s Health Issues* (2014) 24(3):e345-e352. doi: 10.1016/j.whi.2014.02.008

[55] Ai AL, Pappas C, Simonsen E. Risk and protective factors for three major mental health problems among Latino American men nationwide. *Am J Men`s Health* (2015) 9(1):64-75. doi: 10.1177/1557988314528533

[56] Bakhtiari F, Plunkett SW, Alpizar D. Family Qualities, Self-Deprecation, and Depressive Symptoms of Zoroastrian Young Adults in Immigrant Families. *J Immigr Minor Health* (2017) 19(3):645-654. doi: 10.1007/s10903-016-0476-1

[57] Caetano R, Vaeth PA, Canino G. Family cohesion and pride, drinking and alcohol use disorder in Puerto Rico. *Am J Drug Alcohol Abuse* (2017) 43(1):87-94. doi: 10.1080/00952990.2016.1225073

[58] Caetano R, Vaeth PAC, Canino G. Illegal drug use and its correlates in San Juan, Puerto Rico. *Drug Alcohol Depend* (2018a) 185:356-359. doi: 10.1016/j.drugalcdep.2017.12.029

[59] Caetano R, Gruenewald P, Vaeth PAC, Canino G. DSM-5 Alcohol Use Disorder Severity in Puerto Rico: Prevalence, Criteria Profile, and Correlates. Alcohol Clin Exp Res (2018b) 42(2):378-86. doi: 10.1111/acer.13572

[60] Caetano R, Vaeth PAC, Canino G. Comorbidity of Lifetime Alcohol Use Disorder and Major Depressive Disorder in San Juan, Puerto Rico. *J Stud Alcohol Drugs* (2019) 80(5):546-51. doi: 10.15288/jsad.2019.80.546

[61] Cano M, Sánchez M, Rojas P, Ramírez-Ortiz D, Polo KL, Romano E., et al. Alcohol Use Severity Among Adult Hispanic Immigrants: Examining the Roles of Family Cohesion, Social Support, and Gender. *Subst Use Misuse* (2018) 53(4):668-76. doi: 10.1080/10826084.2017.1356333

[62] Cano M, Castro FG, De La Rosa M, Amaro H, Vega WA, Sánchez M, et al. Depressive Symptoms and Resilience among Hispanic Emerging Adults: Examining the Moderating Effects of Mindfulness, Distress Tolerance, Emotion Regulation, Family Cohesion, and Social Support. *Behav Med* (2020) 46(3-4): 245-57. doi: 10.1080/08964289.2020.1712646

[63] Carris MJ, Sheeber L, Howe S. Family rigidity, adolescent problem-solving deficits, and suicidal ideation: a mediational model. J Adolesc (1998) 21(4):459-72. doi: 10.1006/jado.1998.0170

[64] Darghouth S, Brody L, Alegría M. Does Marriage Matter? Marital status, family processes, and psychological distress among Latino men and women. Hispanic J Behav Sci (2015) 37(4):482-502. doi: 10.1177/0739986315606947

[65] Dillon FR, De La Rosa M, Sanchez M, Schwartz SJ. Preimmigration family cohesion and drug/alcohol abuse among recent Latino Immigrants. Family J (2012) 20(3):256-266. doi: 10.1177/1066480712448860

[66] Escobedo P, Allem JP, Baezconde-Garbanati L, Unger JB. Cultural values associated with substance use among Hispanic emerging adults in Southern California. Addict Behav (2018) 77:267-271. doi: 10.1016/j.addbeh.2017.07.018

[67] Guo M, Li S, Liu J, Sun F. Family relations, social connections, and mental health among Latino and Asian older adults. Res Aging (2015) 37(2):123-147. doi: 10.1177/0164027514523298

[68] Guo M, Steinberg NS, Dong X, Tiwari A. A cross-sectional study of coping resources and mental health of Chinese older adults in the United States. Aging Ment Health (2018) 22(11):1448-455. doi: 10.1080/13607863.2017.1364345

[69] Joel Wong Y, Uhm SY, Li P. Asian Americans' family cohesion and suicide ideation: moderating and mediating effects. Am J Orthopsychiatry (2012) 82(3):309-18. doi: 10.1111/j.1939-0025.2012.01170.x

[70] Kwon S. Perceived discrimination, family and spousal relationships, and psychological distress among Asian Americans: Testing mediation and moderation effects. Soc Scienc J (2020) 57(1):26-38*.* doi: 10.1016/j.soscij.2019.01.001

[71] Leong F, Park YS, Kalibatseva Z. Disentangling immigrant status in mental health: psychological protective and risk factors among Latino and Asian American immigrants. Am J Orthopsychiatry (2013) 83(3):361-71. doi: 10.1111/ajop.12020

[72] Litwin H, Shiovitz-Ezra S. Social network type and subjective well-being in a national sample of older Americans. Gerontologist (2011) 51(3):379-88. doi: 10.1093/geront/gnq094

[73] Morimoto Y, Sharma A. Long-Term Outcomes of Verbal Aggression. *J Emotional Abuse* (2004) 4(2):71-99. doi: 10.1300/J135v04n02_04

[74] Park M, Unützer J, Grembowski D. Ethnic and gender variations in the associations between *family cohesion, family conflict, and depression in older Asian and Latino adults.*J Immigr Minor Healt (2014) 16(6):1103-10. doi: 10.1007/s10903-013-9926-1

[75] Priest JB, Denton W. Anxiety Disorders and Latinos: The Role of Family Cohesion and Family Discord. Hisp J Behav Sci (2012) 34(4):557-575. doi: 10.1177/0739986312459258

[76] Rivera FI, Guarnaccia PJ, Mulvaney-Day N, Lin JY, Torres M, Alegria M. Family Cohesion and its Relationship to Psychological Distress among Latino Groups. *Hispanic J Behav Sci* (2008) 30(3):357-78. doi: 10.1177/0739986308318713

[77] Savage JE, Mezuk B. Psychosocial and contextual determinants of alcohol and drug use disorders in the National Latino and Asian American Study. DAD (2014) 139:71-8. doi: 10.1016/j.drugalcdep.2014.03.011

[78] Wang F, Nguyen AW, Lincoln KD, Qin W, Hamler T. The Moderating Role of Race and Ethnicity in the Relationship Between Negative Family Interactions and Mental Health Among Older Adults. Gerontologist (2022) 62 (5):647-84. doi: 10.1093/geront/gnab148

[79] Westrick AC, Sanchez M, Wang W, Cano M, Rojas P, De La Rosa M. Alcohol use severity among recent Latino immigrants: Associations of acculturation, family history of alcohol use and alcohol outcome expectancies. J Ethn Subst Abuse (2021) 22:372-86. doi: 10.1080/15332640.2021.1952126

[80] Gyasi RM, Yeboah AA, Mensah CM, Ouedraogo R, Addae EA. Neighborhood, social isolation, and mental health outcome among older people in Ghana. J Affect Disord (2019) 259:154-63. doi: 10.1016/j.jad.2019.08.024

[81] Xie M, Wang X, Zhang J, Wang Y. Alteration in the psychological status and family environment of pregnant women before and during the COVID-19 pandemic. Int J Gynecol Obstet (2021) 153(1):71-5. doi: 10.1002/ijgo.13575

[82] Diamond GM, Farhat A, Al-Amor, M, Elbedou S, Shelef K, Bar-Hamburger R. Drug and alcohol use among the Bedouin of the Negev: prevalence and psychosocial correlates. Addict Behav (2008) 33(1):143-51. doi: 10.1016/j.addbeh.2007.04.028

[83] Bert F, Lo Moro G, Corradi A, Acampora A, Agodi A, Brunelli L, et al. Prevalence of depressive symptoms among Italian medical students: The multicentre cross-sectional "PRIMES" study. PLoS One (2020b): 15(4):e0231845. doi: 10.1371/journal.pone.0231845

[84] Bert F, Ferrara M, Boietti E, Langiano E, Langiano E, Savatteri A. Depression, suicidal ideation and perceived stress in Italian humanities students: A cross–sectional study. *J Ment Health Phys Health* (2022b): 125(1):256-279

[85] Nam B, Kim JY, De Vylder JE, Song A. Family functioning, resilience, and depression among North Korean refugees. *Psychiatry Res* (2016) 245:451-57. doi: 10.1016/j.psychres.2016.08.063

[86] Yang HJ. Wu JY, Huang SS, Lien MH, Lee TS. Perceived discrimination, family functioning, and depressive symptoms among immigrant women in Taiwan. Arch Women Ment Health (2014) 17(5):359-66. doi: 10.1007/s00737-013-0401-8

[87] Beavers R, Hampson RB. The Beavers Systems Model of Family Functioning. J Fam Ther (2000) 22(2):128-43. doi: 10.1111/1467-6427.00143

[88] Skinner H, Steinhauer P, Sitarenios G. Family assessment measure (FAM) and process model of family functioning. *J Fam Ther* (2000) 22(2):190-210. doi: 10.1111/1467-6427.00146

[89] Baumeister RF, Leary MR. The need to belong: Desire for interpersonal attachments as fundamental human motivation. Psychol Bull (1995) 117(3):497-529. doi: 10.1037/0033-2909.117.3.497

[90] Jetten J Haslam SA, Cruwys T, Greenaway KH, Haslam V, Steffens NK. Advancing the social identity approach to health and well-being: Progressing the social cure research agenda. *Europ J Soc Psychol* (2017) 47(7):789-802. doi: 10.1002/ejsp.2333

[91] Furstenberg FF, Kaplan SB. Social capital and the family. In: The Blackwell companion to the sociology of families. Scott J, Treas J, Richards M. (eds.)Blackwell, Malden, USA 2004. 218-232.

[92] Saeri T, Cruwys FK, Barlow S, Stronge S, Sibley CG. Social connectedness improves public mental health: Investigating bidirectional relationships in the New Zealand attitudes and values survey. Aust NZ J Psychiatry (2018) 52(4):365-74. doi: 10.1177/0004867417723990
